# Isolation of Microcystins from the Cyanobacterium *Planktothrix rubescens* Strain No80

**DOI:** 10.1007/s13659-013-0001-3

**Published:** 2014-02-18

**Authors:** Timo H. J. Niedermeyer, Peter Schmieder, Rainer Kurmayer

**Affiliations:** 1 Cyano Biotech GmbH, Magnusstr. 11, 12489 Berlin, Germany; 2 Interfaculty Institute for Microbiology and Infection Medicine Tübingen, Eberhard Karls University Tübingen, Auf der Morgenstelle 28, 72076 Tübingen, Germany; 3 Leibniz Institut für Molekulare Pharmakologie, Robert-Rössle-Str. 10, 13125 Berlin, Germany; 4 Research Institute for Limnology, University of Innsbruck, Mondseestrasse 9, 5310 Mondsee, Austria

**Keywords:** *Planktothrix*, Cyanobacteria, Microcystins, Protein phosphatase inhibition

## Abstract

Three minor microcystins have been isolated from a *Planktothrix rubescens* strain. Their structures have been elucidated by one- and two-dimensional NMR spectroscopy and high-resolution tandem mass spectrometry as the compounds [Asp^3^,(*E*)-Dhb^7^]MC-LY (**1**), [Asp^3^,(*E*)-Dhb^7^]MC-HtyW (**2**), and [Asp^3^,(*E*)-Dhb^7^]MC-LW (**3**). The amino acids found at the variable positions 2 and 4 of the microcystin core structure are in accordance with the predicted amino acid substrate activation selectivities of the non-ribosomal peptide synthetases McyA and McyB described earlier for this strain. All structural microcystin variants produced by this strain were shown to inhibit protein phosphatase 1 in the nanomolar range.

**Electronic supplementary material:**

The online version of this article (doi:10.1007/s13659-013-0001-3) contains supplementary material, which is available to authorized users.

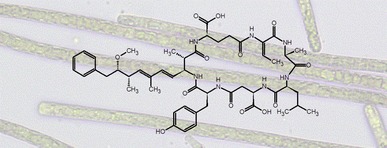

Among toxins produced by cyanobacteria in freshwater and estuarine systems, the microcystins (MCs) are the most frequent. MCs are cyclic heptapeptides. The most common core structure of these compounds is cyclo(-d-Ala^1^-l-X^2^-d-MeAsp^3^-l-Z^4^-Adda^5^-d-Glu^6^-Mdha^7^), where X and Z are variable l-amino acids, d-MeAsp is d-*erythro*-ß-iso-aspartic acid, Adda is (2*S*,3*S*,8*S*,9*S*)-3-amino-9-methoxy-2,6,8-trimethyl-10-phenyldeca-4,6-dienoic acid, and Mdha is *N*-methyl-dehydroalanine [1]. More than 90 different natural MC congeners containing different amino acids especially at the variable positions 2 and 4, but also at the other positions 1, 3, 5, 6, and 7, have been described from both field and isolated strain samples [2, 3]. The most common variants (MC-RR, MC-YR, and MC-LR) feature either Arg, Tyr, or Leu in position 2 of the molecule, while Arg is predominant at position 4. *Planktothrix* strain No80 does not produce these common MC-variants, but rather the homotyrosine (Hty) containing congeners [Asp^3^,(*E*)-Dhb^7^]MC-HtyY and [Asp^3^,(*E*)-Dhb^7^]MC-HtyHty instead [4]. Notably, the genotypes that are able to synthesize these MC variants were only found in one habitat, and it was concluded that relaxed selection constrains paired with spatial isolation led to the appearance of this unique MC producer [5, 6]. Since this strain also produces several minor MC structural variants in addition to the most dominant compounds [4], we were interested in their structural elucidation. Thus three compounds featuring the typical UV spectra of MCs containing Tyr or Trp have been isolated (**1**–**3**), and their structures have been elucidated. As it is known that MCs are potent inhibitors of protein serine/threonine phosphatases (PP) 1 and PP2A [7], the PP inhibitory activity of all the structural variants has been tested using MC-LR as reference compound.

## Results and Discussion

*Planktothrix rubescens* strain No80 was grown in laboratory culture, and the dried cell biomass was extracted using aqueous methanol. Compounds (**1**–**3**) were isolated after fractionation of the crude extract on C_18_ solid-phase extraction cartridges followed by semi-preparative HPLC. The three compounds were obtained as amorphous white powders.

Tandem MS data confirmed that the compounds were MC variants, as for all three substances significant peaks at *m*/*z* 375.19 (z_3[7|1][5–7]_ − C_9_H_10_O ion) as well as fragments at *m*/*z* 135 and the corresponding [M − C_9_H_10_O]^+^ fragments could be observed, ions characteristic for MCs containing the Adda substructure [8–10]. To facilitate structure elucidation, a Python script (see ESM) has been written to calculate molecular masses of possible MC variants based upon the residues found in microcystins isolated to date, taking into account monomers commonly observed for MCs from *Planktothrix* and *Oscillatoria* (e.g. Ala at position 1, Asp at position 3 of the MCs found in these genera) as well as the predicted amino acid substrate activation selectivities of the non-ribosomal peptide synthetase McyA found in *P. rubescens* strain No80 (Dhb at position 7) [4]. The script output gave indications which MC structures might match with the observed molecular masses of the three compounds.

HRMS data for compound **1** were consistent with a molecular formula of C_51_H_69_N_7_O_13_ (found *m*/*z* 988.5052, calcd. 988.5026 for [M + H]^+^, Δ 2.6 ppm). The ^1^H NMR spectrum of **1** in *d*_6_-DMSO showed close similarities to those of other MCs isolated from *Planktothrix* [4]. The NH-CH_α_ cross peaks in the TOCSY spectrum served as the basis for the identification of the individual spin systems, and in conjunction with HMQC and HMBC data, the seven monomers in **1** could unambiguously be identified as Ala, Leu, Asp, Tyr, Adda, Glu, and 2-amino-2-butenoic acid (Dhb) (Table 1). As for the other MCs isolated from this strain [4], ROESY data supported the conventional all-E configurations of the Adda double bonds. The presence of the Dhb residue was revealed by a methyl doublet and an olefinic proton quartet resonating at *δ* 1.87 and at *δ* 5.61, typical for (*E*)-Dhb [4]. Leu and Tyr are the variable amino acids incorporated into positions 2 and 4 of this MC congener. The location of these two amino acids was established on the basis of ROESY NMR and tandem MS data. Various ROESY correlations between Ala and Leu protons were detected. Furthermore, fragments at *m*/*z* 157 and 185 observed in the tandem mass spectrum of **1** confirmed the direct proximity of Ala and Leu (a- and b-ions of the fragment Ala-Leu), proving that Leu is located at position 2 of this MC. ROESY correlations between Adda protons and Tyr protons support the close proximity of these two monomers, thus Tyr is situated at position 4. In addition to several ROESY correlations as indicated in Table 1, the postulated sequence of **1** was completely confirmed with high confidence by tandem MS. Using the software mMass to assign the peaks observed in the IT-TOF tandem mass spectrum [10], all signals could be assigned within a mass accuracy of 0.01 Da (for more information see ESM). The structure of **1** could thus be determined as [Asp^3^,(*E*)-Dhb^7^]MC-LY. This compound has been tentatively reported from *Planktothrix* before, based on HPLC-MS^2^ analyses and thiol derivatization [11].

**Table 1:** ^1^H (750 MHz, *d*_6_-DMSO) and ^13^C NMR Spectroscopic Assignments for [Asp^3^,(*E*)-Dhb^7^]MC-LY (**1**)

Unit	Position	*δ*_C_	*δ*_H_ (*J* in Hz)	HMBC^a^	ROESY
Adda	1	173.7			
	2	41.9	3.07, m		3
	3	54.4	4.29, m	11	2, 5, 11, NH
	4	126.5	5.32, m		3, 5, 7, 11, 12, NH, Tyr-5/9, Tyr-6/8
	5	135.5	6.13, d (15.3)	3, 7, 12	3, 4, 7, 11, 12, 16/20, 17/19, Tyr-5/9, Tyr-6/8
	6	131.6			
	7	134.8	5.47, d (9.2)	5, 12, 13	4, 5, 8, 9, 10, 13, 14, 16/20, 17/19, Tyr-5/9, Tyr-6/8
	8	35.1	2.57, m	6, 7, 9, 13	7, 9, 10a/b, 12, 13, 14
	9	85.5	3.27, m	7, 8, 10, 13, 14, 15	7, 8, 10, 13
	10a	36.7	2.76, dd (13.7, 4.6)	8, 9, 15, 16/20	7, 8, 9, 13, 14, 16/20
	10b		2.69, dd (13.7, 6.9)	8, 9, 15, 16/20	7, 8, 9, 13, 14, 16/20
	11	15.6	0.88, br. d (3.8)	1, 3	3, 4, 5
	12	12.2	1.57, s	5, 6, 7	4, 5, 8, 14
	13	15.7	0.99, d (6.9)	7, 8, 9	7, 8, 10a/b, 14
	14	57.0	3.20, s	9	7, 8, 10a/b, 12, 13, 16/20
	15	138.9			
	16/20	129.0	7.20, m	10, 15, 16/20, 17/19, 18	5, 7, 10a/b, 14
	17/19	127.8	7.27, m	15, 16/20, 17/19, 18	5, 7, 10a/b, 14
	18	125.5	7.17, m	15, 16/20, 17/19	
	NH		8.19, br. s		3, 4, Tyr-2
Glu	1	171.6			
	2	51.6	4.25, m		3a, 4b
	3a	25.2	1.91, m	1, 5	3a
	3b		1.75, m	1, 5	2, 3b, 4b
	4a	32.0	2.44, m	5	2, 3a, Dhb-NH
	4b		2.04, m		Dhb-NH
	5	170.9			
	NH		9.25, br. s		
Dhb	1	162.6			
	2	130.6			
	3	120.4	5.61, q (6.9)	1, 2, 4	4, NH, Tyr-6/8
	4	12.7	1.87, d (6.9)	1, 2, 3	3, Tyr-6/8
	NH		10.07, br. s		3, 4, Tyr-2, Glu-4a/4b
Ala	1	172.2			
	2	46.2	4.37, m	Dhb-1, 1, 3	3, NH, Leu-5, Leu-6, Leu-NH
	3	15.9	0.69, br. d		2, NH, Tyr-5/9, Tyr-6/8, Tyr-OH
	NH		7.06, br. s		2, 3, Leu-NH
Leu	1	170.6			
	2	53.4	3.89, m		3b, 4, 6, NH
	3a	38.4	1.76, m		2, 3a, 4, NH
	3b		1.42, m	1	3b, NH
	4	23.8	1.64, m		2, 3b, NH
	5	22.7	0.84, d (6.1)	3, 4, 6	Ala-2
	6	20.7	0.78, d (6.1)	3, 4, 5	2, NH, Ala-2
	NH		7.98, br. s		2, 3a/b, 4, 6, Ala-2, Ala-NH
Asp	1	170.0			
	2	50.2	4.16, m		
	3a	37.6	2.75, m		3a
	3b		1.52, m		3b, Tyr-NH
	4	n.o.			
	NH		7.78, br. s		
Tyr	1	173.0			
	2	52.8	4.69, m		Adda-NH, 3a/b, 5/9, NH
	3a	35.4	3.30, m	1	2, 3a, 5/9, NH
	3b		2.26, m		2, 3b, 5/9
	4	129.2			
	5/9	128.3	6.92, d (7.6)	3, 4, 5/9, 6/8, 7	2, 3a/b, Ala-3
	6/8	114.3	6.43, d (7.6)	5/8, 7	Ala-3
	7	155.0			
	NH		8.52, br. s		2, 3b, Asp-3a
	OH		8.99, br. s		6/8, Ala-3

^13^C chemical shifts obtained from HMQC and HMBC spectra (^1^H frequency 600 MHz, in *d*_6_-DMSO)

*n.o.* not observed

^a^HMBC correlations are stated from proton to the indicated carbon

 HRMS data of compound **2** supported the molecular formula C_57_H_70_N_8_O_13_ (found *m*/*z* 1075.5133, calcd. 1075.5135 for [M + H]^+^, Δ 0.2 ppm). Its ^1^H NMR spectrum resembled that of **1** concerning the prominent signals of Adda and Dhb. However, there were also striking differences observed: While the Leu methyl group resonances were missing, an additional NH proton at very low field (10.60 ppm) as well as additional signals in the aromatic region indicated the presence of Trp and the absence of Leu. Closer examination of the TOCSY and HMBC spectra, however, revealed that Hty, rather than the Tyr found in **1**, was present in this MC congener (NMR data see Table 2). To establish the sequence of **2**, again ROESY and tandem MS were used. ROESY data clearly showed the proximity of Adda and Trp, and of Ala and Hty. ROESY data also showed a rather intriguing conformation of **2**. Both Trp and Hty showed ROESY correlations with Ala, implying that these flexible side chains seem to cluster in this part of the molecule. As with **1**, the sequence of **2** has been confirmed using mMass. Here, an a-ion at *m*/*z* 221 confirmed the sequence Ala-Hty, and most of the peaks of the tandem mass spectrum could be annotated based on the postulated sequence. Taking all data into account, **2** was identified as [Asp^3^,(*E*)-Dhb^7^]MC-HtyW. The presence of both Hty and Trp make this MC congener one of those with the highest molecular weight.

**Table 2:** ^1^H (750 MHz, *d*_6_-DMSO) and ^13^C NMR Spectroscopic Assignments for [Asp^3^,(*E*)-Dhb^7^]MC-HtyW (**2**)

Unit	Position	δ_C_	δ_H_ (*J* in Hz)	HMBC^a^	ROESY
Adda	1	174.7			
	2	42.1	3.03, m	4	11, Trp-3b
	3	53.9	4.35, m	1	5, 11, NH
	4	126.1	5.35, m		5, 11, 12, NH
	5	135.1	6.16, d (15.3)	3, 6, 12, 7	3, 4, 7, 11
	6	131.8			
	7	134.5	5.50, d (9.2)	5, 12	5, 8, 9, 10a/b, 13
	8	35.0	2.59, m	6, 7, 9, 13	7, 9, 12, 13, 14
	9	85.6	3.28, m	7, 8, 11, 12, 14	7, 8, 10a/b, 13
	10a	36.5	2.76, dd (13.7, 4.6)	8, 9, 15, 16/20	7, 9, 12, 13, 16/20
	10b		2.70, dd (13.7, 7.6)	8, 9, 15, 16/20	7, 9, 13, 16/20
	11	15.6	0.90, d (4.6)	1, 2, 3	2, 3, 4, 5
	12	12.0	1.58, s	5, 6, 7	4, 8, 10a, 13
	13	15.5	1.00, d (6.1)	7, 8, 9	7, 8, 9, 10a/b, 12, 14
	14	56.9	3.21, s	9	8, 13
	15	138.9			
	16/20	128.9	7.21, m	15, 16/20, 17/19, 20	10a/b
	17/19	127.7	7.27, m	15, 16/20, 17/19, 18	
	18	125.3	7.18, m	15, 16/20, 17/19	
	NH		8.15, br. s		3, 4, Trp-2
Glu	1	171.2			
	2	49.8	4.24, m	4	
	3a	24.1	1.91, m	1	
	3b		1.74, m	5	
	4a	31.5	2.41, m	5	
	4b		2.06, m		Dhb-NH
	5	171.7			
	NH		9.24, br. s		
Dhb	1	162.7			
	2	130.4			
	3	120.9	5.62, q (6.1)	1, 2, 4	4, NH
	4	12.6	1.86, d (6.1)	2, 3	3, NH, Trp-5, Hty-2, Hty-3b
	NH		10.00, br. s		3, 4, Glu-4b
Ala	1	n.o.			
	2	45.9	4.19, m		Hty-NH
	3	15.3	0.43, br. d		
	NH		6.95, br. s		
Hty	1	n.o.			
	2	53.9	3.76, m		3b, 4b, NH, Dhb-4
	3a	32.1	1.99, m	5	NH
	3b		1.83, m		2, 7/9, Dhb-4
	4a	30.2	2.55, m	6/10	4b, NH
	4b		2.35, m		2, 4a, 6/10, NH
	5	129.4			
	6/10	129.0	6.93, d (7.6)	4, 6/10, 8	4b, 7/9
	7/9	114.5	6.61, d (7.6)	6/10, 7/9, 8	3b, 6/10, Trp-10
	8	155.0			
	NH		8.06, br. s		2, 3a, 4a/b, Ala-2
	OH		9.06, br. s		
Asp	1	174.3			
	2	51.8	4.23, m		
	3a	37.7	2.80, m	1	3b
	3b		1.48, m		3a
	4	n.o.			
	NH		7.77, br. s		
Trp	1	173.2			
	2	51.2	4.78, m	3	3a, 7, Adda-NH
	3a	26.4	3.51, m		2, 3b
	3b		2.59, m	1	3a, NH_amide_, Adda-2
	4	110.9			
	5	118.0	6.79, m	4, 6	7, Dhb-4
	6	126.7			
	7	117.8	7.54, d (7.6)	4, 6, 9, 11	2, 5, 8
	8	122.3	6.94, m	6, 10	7
	9	120.2	6.96, m	7, 11	10
	10	110.7	7.24, d (7.6)	6, 7	9, Hty-7/9
	11	136.0			
	NH_amide_		8.58, br. s		2, 3b
	NH_indole_		10.60, br. s	6, 10	

^13^C chemical shifts obtained from HMQC and HMBC spectra (^1^H frequency 600 MHz, in d_6_-DMSO)

*n.o.* not observed

^a^HMBC correlations are stated from proton to the indicated carbon

**Fig. 1:**
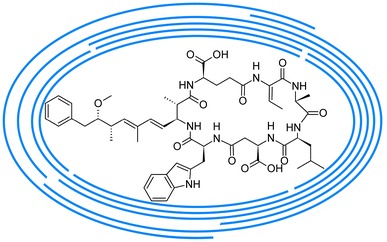
Interlaced b-ions observed in the tandem MS spectrum of **3** (for details on the observed fragments see ESM)

HRMS of **3** showed a quasi-molecular ion at *m*/*z* 1011.5172, corresponding to the molecular formula C_53_H_70_N_8_O_12_ (calcd. 1011.5186 for [M + H]^+^, Δ − 1.4 ppm). The ^1^H NMR spectrum showed similarities with the spectra of both **1** and **2**: The typical NH and aromatic signals of a Trp were observed as in **2**, and methyl resonances as in **1** indicated the presence of Leu. However, the aromatic signals of the Tyr/Hty moiety were missing. COSY correlations confirmed the presence of Leu and Trp as well as of Adda and Dhb (Table 3). As only small amounts of **3** could be isolated, the carbon chemical shifts could not be deduced from HSQC or HMBC spectra. Sequence elucidation was thus based on tandem MS data, a common approach for MCs [9, 12, 13]. Again, the a- and b-ion fragments for Ala-Leu could be observed, showing that Leu was present at position 2 of this MC. The theoretical fragments calculated for the postulated sequence match well with the observed fragments, and the observed b-ions can be interlaced to cover the complete postulated sequence (Fig. 1). Furthermore, the ^1^H chemical shifts of Trp and Leu in **3** were almost identical with those of the respective monomers in **1** and **2**, supporting the assumption that these monomers were located in the same positions as Leu in **1** and Trp in **2**. The structure of **3** can thus be postulated to be [Asp^3^,(*E*)-Dhb^7^]MC-LW.

**Table 3:** Selected ^1^H NMR Spectroscopic Assignments (750 MHz, *d*_6_-DMSO) for [Asp^3^,(*E*)-Dhb^7^]MC-LW (**3**)

Monomer	Position	δ_H_ (*J* in Hz)	COSY
Adda	2	3.00, m	3, 11
	3	4.34, m	2, 4
	4	5.32, m	3, 5
	5	6.17, d (15.3)	
	7	5.51, d (9.2)	8, 12
	8	2.59, m	7, 9, 13
	9	3.27, m	8, 10a/b
	10a	2.76, m	9
	10b	2.69, m	9
	11	0.90, d (4.6)	2
	12	1.58, s	7
	13	1.00, d (6.9)	8
	14	3.20, s	
	16/20	7.21, m	17/19
	17/19	7.27, m	16/20, 18
	18	7.19, m	17/19
	NH	8.19, br.	
Dhb	3	5.61, m	4
	4	1.88, m	3
	NH	9.95, br.	
Leu	2	3.88, m	3a/b, NH
	3a	1.77, m	2, 3b
	3b	1.39, m	2, 3a, 4
	4	1.63, m	3b, 5, 6
	5	0.82, m	4
	6	0.75, d (6.1)	4
	NH	7.92, br.	2
Trp	2	4.80, m	3b, NH_amide_
	3a	3.48, m	3b
	3b	2.62, m	2, 3a
	5	6.77, m	7, 8
	7	7.54, d (7.6)	5
	8	6.94, m	5
	9	6.96, m	10
	10	7.23, d (7.6)	9
	NH_amide_	8.59, br.	2
	NH_indole_	10.59, br.

As has been shown previously [14], the adenylation domains (A-domains) encoded by the *mcy*BA1 and *mcy*CA genes that are part of the MC (*mcy*) synthetase gene cluster are responsible for the recognition and activation of amino acids at the variable positions 2 and 4, resp. Comparing a larger number of strains, within *mcy*BA1 an exclusive correlation between a certain *mcy*BA1 genotype and the occurrence of Hty or Leu in position 2 of the molecule with simultaneous absence of Arg at this position was recognized [6]. The *mcy*BA1 genotype of *Planktothrix* strain No80 was shown to fit exactly to this genotype [4]. Surprisingly, *mcy*CA showed much less nucleotide variation compared to *mcy*BA1, and only a few point mutations could be related to the presence of Tyr/Hty in position 4 of the molecule [4]. It is interesting to note that the same *Mcy*CA domain, which has no precedent in the NRPS database [15, 16], apparently is able to activate an additional aromatic amino acid substrate, Trp, as observed in compounds **2** and **3**.

MC biosynthesis in *Planktothrix* has been studied in great detail, and it is known that different MC congeners synthesized by an individual strain are products of the same MC synthetase [17]. Amino acid variability in MC congeners is due to multispecific domains that allow for incorporation of different amino acids rather than different biosynthesis enzymes [17]. For two MCs isolated from *P. rubescens* strain No80, [Asp^3^,(*E*)-Dhb^7^]MC-HtyHty and [Asp^3^,(*E*)-Dhb^7^]MC-HtyY, the absolute configuration of the residues d-Ala, d-Glu, d-Asp, l-Tyr and l-Hty has been determined by chiral GC after hydrolysis and trifluoroacetylation [4]. Because all the MC variants found in this strain are synthesized by the same synthetase, it can safely be assumed that the amino acids in **1**, **2**, and **3** have the same configuration as those in the first two isolated MCs.

The protein phosphatase (PP) inhibition assay revealed that all MC structural variants inhibited PP-1 in the nanomolar range (Table 4). All IC_50_ estimates were comparable to the inhibitory activity of MC-LR, confirming that the residues in position 2, 3, 4, and 7 of the MCs have only little influence on PP-1 inhibition potency, which is mainly due to the Adda^5^-Glu^6^ substructure [18–21].

**Table 4:** PP-1 inhibitory activity of five [d-Asp^3^,Dhb^7^]-MC structural variants produced by *Planktothrix* strain No80 and MC-LR

	IC_50_ (nM)	Fitting error (%)	*R*^2^ of fit
MC-LR	2.0	16.5	0.9923
[Asp^3^,(*E*)-Dhb^7^]MC-HtyY	2.9	16.0	0.9867
[Asp^3^,(*E*)-Dhb^7^]MC-HtyHty	3.2	11.1	0.9963
[Asp^3^,(*E*)-Dhb^7^]MC-LY (**1**)	3.8	13.6	0.9953
[Asp^3^,(*E*)-Dhb^7^]MC-HtyW (**2**)	5.0	10.1	0.9970
[Asp^3^,(*E*)-Dhb^7^]MC-LW (**3**)	5.4	15.2	0.9947

## Experimental Section

### General Experimental Procedures

Samples for NMR spectroscopy were dissolved in 600 μL *d*_6_-DMSO to yield concentrations of 1.7 mM (**1**), 1.1 mM (**2**), and 0.5 mM (**3**). NMR spectra were recorded at 600 or 750 MHz (^1^H frequency) on Bruker AV-III spectrometers using cryogenically cooled 5 mm TCI-triple resonance probes equipped with one-axis self-shielded gradients. All homonuclear two-dimensional spectra (DQF-COSY [22], TOCSY [23, 24], ROESY [25, 26] were recorded using 2,048 × 512 complex data points. DQF-COSYs and TOCSYs were recorded using 8 scans, the ROESY using 64 scans, the TOCSYs with a mixing time of 80 ms, the ROESYs with a mixing time of 100 ms. Low-power irradiation at the frequency of the water resonance was used to remove signals from residual water. Heteronuclear two-dimensional ^13^C-HMQC [27] and the ^13^C-DEPT-HMQC [28] spectra were recorded using 512 × 512 complex data points using 96 scans, while the ^13^C-HMQC-TOCSY [29] spectra were recorded with 512 × 256 complex data points using 96 scans and a mixing time of 80 ms. All the above heteronuclear spectra were recorded using a BIRD pulse for suppression of protons bound to ^12^C [30]. A gradient-enhanced-^13^C-HMBC [31] was recorded with 2048 × 512 complex data points using 160 scans. Spectra were referenced indirectly to tetramethylsilane via the residual signals of *d*_6_-DMSO (2.50 ppm for ^1^H, 39.5 ppm for ^13^C). ^13^C chemical shifts were extracted from the two-dimensional ^13^C-spectra. IT-TOF MS data have been acquired using an HPLC coupled to an IT-TOF mass spectrometer (Shimadzu Europe GmbH, Duisburg, Germany) with electrospray ionization in positive mode and were evaluated using the vendor’s software LCMSSolution version 3.60.361 with Formula Predictor version 1.13. The compounds were separated on a Kinetex C_18_ column (2.6 μm, 100 × 3 mm, phenomenex, Torrance, USA) using a gradient ranging from 5 to 80 % CH_3_CN in water over 25 min (0.1 % formic acid added as modifier). Precursor ions corresponding to [M + H]^+^ were isolated in the ion trap, fragmented by collision induced dissociation (CID) using argon as collision gas (collision energy set to 150 %, collision gas to 100 %, and q(Frequency) to 45.0 kHz), and separated in the TOF analyzer. MS/MS scans were averaged and converted to the mzXML format using the vendor’s software. For the calculation of sum formulae, the monoisotopic mass averaged from at least three scans has been used (resolution 10000, external calibration using sodium TFA ions clusters immediately prior to analysis). QTOF MS data were acquired using an ABSciex Qstar XL equipped with a nano electrospray source. Precursor ions were isolated with low quadrupole resolution. Fragmentation energies were set to automatic (rolling collision energy).

### Cyanobacterial Material

*Planktothrix rubescens* strain No80 has been isolated from Lake Schwarzensee (Upper Austria) [5]. It was classified as *P. rubescens* on the basis of PCR analysis and sequencing of various marker genes [32], and has been deposited under this accession number in the culture collection of the Institute for Limnology in Mondsee, Austria. The strain was cultivated in BG11 medium [33] at 20 °C under continuous light (60–80 μmol m^−2^ s^−1^) in 20 L scale photobioreactors and harvested semi-continuously over a period of several weeks.

### Extraction and Isolation

Cyanobacterial cells were harvested and lyophilized. 78.0 g dry biomass were suspended in 50 % MeOH (v/v), treated with an ultrasonication rod (Bandelin, Berlin, Germany) and extracted on a shaker for 30 min. After centrifugation the biomass was subsequently extracted using 80 % MeOH (v/v). The extracts were combined and dried *in vacuo*, yielding 17.2 g of crude extract. 14 g of the crude extract were fractionated using a C_18_ cartridge (VersaPak, 40–75 μm, 40 × 150 mm) on a VersaFlash system (supelco, Bellefonte, USA). A step gradient of 20, 40, 60 and 80 % MeOH in water (v/v), each 700 mL, has been used at a flow rate of 20 mL/min. The 60 % MeOH (v/v) fraction containing the MCs was dried *in vacuo*. After reconstitution, this fraction was subjected to semi-preparative HPLC on a SymmetryShield RP18, 5 μm, 10 × 250 mm column (Waters, Milford, USA) using a linear step gradient of aqueous CH_3_CN (with 0.025 % v/v TFA) at 2.5 mL/min, starting with 20 % CH_3_CN, increasing to 32.5 % CH_3_CN in 20 min, stepping to 42.5 % CH_3_CN and increasing to 55 % within 25 min. About 1.2 mg of compound **1**, 1.0 mg of compound **2**, and 0.6 mg of compound **3** were isolated. The compounds eluted at 24.6 min (**1**), 27.6 min (**2**), and 28.6 min (**3**) under analytical HPLC conditions as described previously [5].

**[Asp**^**3**^**,(*E*)-Dhb**^**7**^**]MC-LY** (**1**): white, amorphous powder; UV (H_2_O/MeCN/TFA) *λ*_max_ 232 nm; ^1^H and ^13^C NMR data see Table 1; HRESIMS *m*/*z* [M + H]^+^ 988.5052 (calcd for C_51_H_69_N_7_O_13_, 988.5026).

**[Asp**^**3**^**,(*E*)-Dhb**^**7**^**]MC-HtyW** (**2**): white, amorphous powder; UV (H_2_O/MeCN/TFA) *λ*_max_ 222 nm; ^1^H and ^13^C NMR data see Table 2; HRESIMS *m*/*z* [M + H]^+^ 1075.5133 (calcd for C_57_H_70_N_8_O_13_, 1075.5135).

**[Asp**^**3**^**,(*E*)-Dhb**^**7**^**]MC-LW** (**3**): white, amorphous powder; UV (H_2_O/MeCN/TFA) *λ*_max_ 222 nm; ^1^H NMR data see Table 3; HRESIMS *m*/*z* [M + H]^+^ 1011.5172 (calcd for C_53_H_70_N_8_O_12_, 1011.5186).

### Protein Phosphatase Inhibition Assay

A colorimetric protein phosphatase inhibition assay [34] was used to determine the IC_50_ of all the MC structural variants produced by *Planktothrix* strain No80. The catalytic subunit (α-isoform) of protein phosphatase 1 (PP-1) from rabbit muscle (Sigma, Vienna, Austria) was diluted according to the manufacturer’s instructions. The assay was carried out in 96-well microtiter plates. 10 μL of enzyme dilution (0.05 U of PP-1) were added to 10 μL of compound solution (dilution series; dissolved in 50 % (v/v) methanol). The enzyme was activated for 5 min at 37 °C, and the reaction was started by adding 180 μL of reaction buffer (25 mM imidazole, pH 7.4, 0.1 mg mL^−1^ BSA, 1 mM DTT, 50 mM NaCl, 25 mM p-nitrophenyl phosphate). To determine 100 % PP-1 activity, 10 μL of 50 % (v/v) aqueous methanol was used instead of compound solution. For the 0 % activity control, water was added instead of PP-1 enzyme dilution. MC-LR was used as a reference and gave IC_50_ values comparable to those reported previously for this compound [21, 34–36]. MC congeners were quantified by measuring the adsorption at 240 and 230 nm and using the molar extinction coefficient of MC-YR (*ε* = 41,100) [37] for all MC variants synthesized by strain No80. Incubation was for 40 min at 37 °C and the microtiter plates were read at 405 nm with an ELISA plate reader (Jupiter, Asys Hitech, Eugendorf, Austria). All MC variants were tested in triplicate and the experimental data were evaluated following current recommendations [38]. IC_50_ values were interpolated from a four parameter logistic model fit using the software EC_50_^calculator^ (see ESM for more details).

## Supporting Information

All NMR and MS raw data as well as the Python script used to calculate the composition of possible MC congeners are available free of charge for download at http://dx.doi.org/10.6084/m9.figshare.706367. Detailed evaluation and annotation of the tandem mass spectra, 1H NMR spectra, output of the Python script, and information on the IC_50_ calculation, are available in the online version of this article.

## Electronic supplementary material

Below is the link to the electronic supplementary material.

All NMR and MS raw data as well as the Python script used to calculate the composition of possible MC congeners are available free of charge for download at http://dx.doi.org/10.6084/m9.figshare.706367. Detailed evaluation and annotation of the tandem mass spectra, ^1^H NMR spectra, output of the Python script, and information on the IC_50_ calculation, are available in the online version of this article. (PDF 8671 kb)
