# Supplementary material for: Isolation of Microcystins from the Cyanobacterium Planktothrix rubescens Strain No80
Source: Nat Prod Bioprospect. 2014 Feb 18;4(1):37–45. doi: 10.1007/s13659-013-0001-3 (PMC3956966; doi:10.1007/s13659-013-0001-3)

Supporting Information for the manuscript

Isolation of three microcystins from the cyanobacterium *Planktothrix rubescens* strain No80

Timo H. J. Niedermeyer, Peter Schmieder, Rainer Kurmayer

Evaluation and annotation of the tandem mass spectra; NMR data of 1-3 2
[Asp^3,(*E*)-Dhb^7]-MC-LY (1) 2
[Asp^3,(*E*)-Dhb^7]-MC-HtyW (2) 8
[Asp^3,(*E*)-Dhb^7]-MC-LW (3) 14
Python Script and its output 18
Script 18
Output 19
EC_{50} calculation 20
Script 20
Output 24
Typical UV spectra of microcystins 25

Evaluation and annotation of the tandem mass spectra; NMR data of 1 – 3

[Asp^3,(*E*)-Dhb^7]-MC-LY (1)

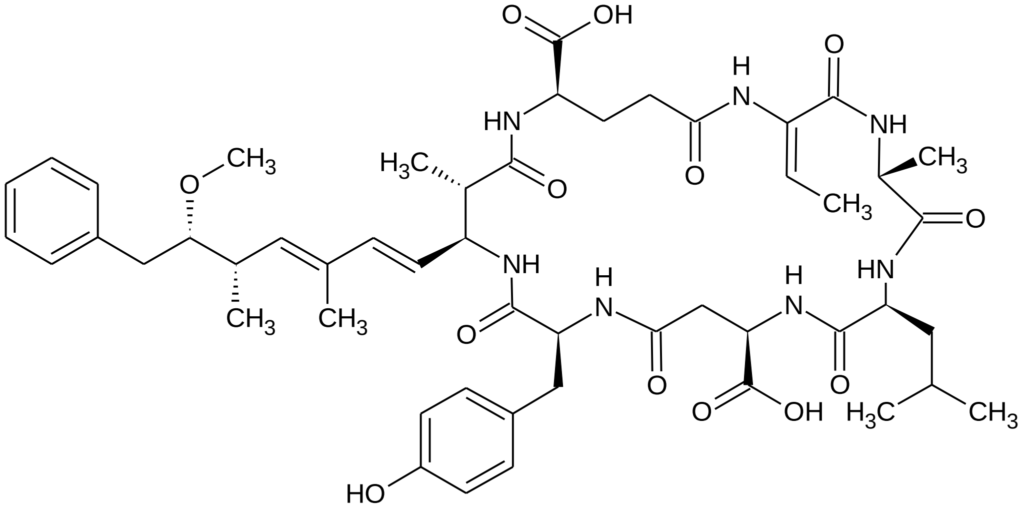

D-Ala | Leu | D-Asp | Tyr | Adda | D-Glu | Dhb

Formula (M)	Ion	Meas. m/z	Pred. m/z	Diff (mDa)	Diff (ppm)	Iso Score
C51 H69 N7 O13	[M+H]+	988.5052	988.5026	2.6	2.63	80.53

IT-TOF tandem MS

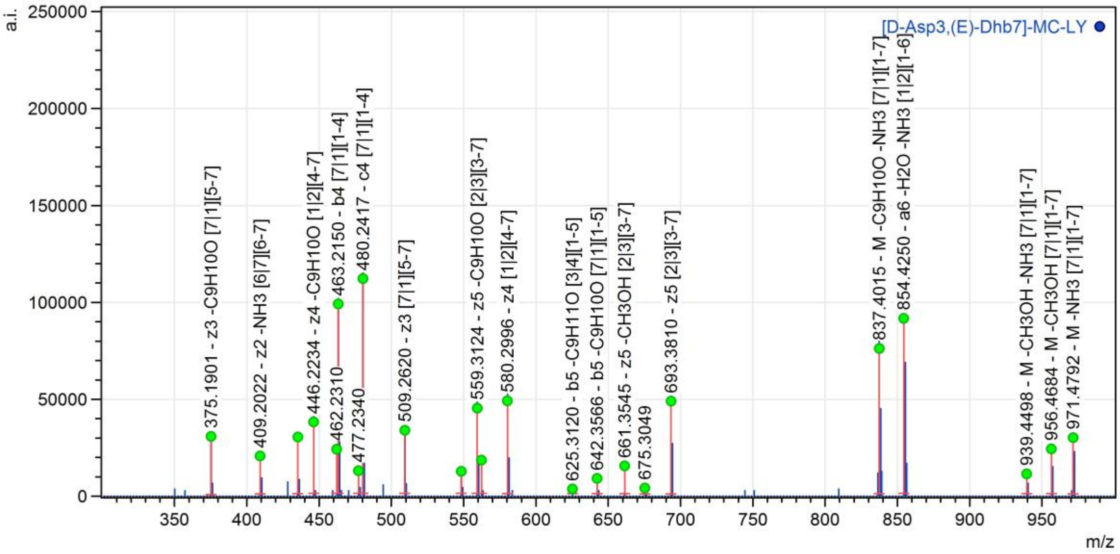

Length	Mo. Mass	Av. Mass	Coverage	Matched Int.
7 (Cyclic)	987.4953	988.1349	100.0 %	100.0 %

Meas. m/z	Calc. m/z	δ (Da)	δ (ppm)	Rel. Int. (%)	Annotation	Formula
375.1901	375.1914	-0.0013	-3.6	27.07	z3 -C9H10O [7|1][5-7]	C20H26N2O5

375.1901	375.1914	-0.0013	-3.6	27.07	b3 -C9H10O -NH3 [4|5][1-3]	C20H26N2O5
409.2022	409.2010	0.0012	3.0	17.69	z2 -NH3 [6|7][6-7]	C25H28O5
435.2201	435.2238	-0.0038	-8.6	26.35	a4 [7|1][1-4]	C21H30N4O6
446.2234	446.2286	-0.0052	-11.6	33.35	z4 -C9H10O [1|2][4-7]	C23H31N3O6
446.2234	446.2286	-0.0052	-11.6	33.35	b4 -C9H10O -NH3 [4|5][1-4]	C23H31N3O6
462.2310	462.2347	-0.0037	-8.1	20.60	c4 -H2O [7|1][1-4]	C22H31N5O6
463.2150	463.2187	-0.0037	-7.9	88.17	b4 [7|1][1-4]	C22H30N4O7
477.2340	477.2384	-0.0044	-9.2	10.62	z3 -CH3OH [7|1][5-7]	C28H32N2O5
477.2340	477.2384	-0.0044	-9.2	10.62	b3 -CH3OH -NH3 [4|5][1-3]	C28H32N2O5
480.2417	480.2453	-0.0036	-7.5	100.00	c4 [7|1][1-4]	C22H33N5O7
509.2620	509.2646	-0.0026	-5.1	29.31	b3 -NH3 [4|5][1-3]	C29H36N2O6
509.2620	509.2646	-0.0026	-5.1	29.31	z3 [7|1][5-7]	C29H36N2O6
548.2773	548.2755	0.0018	3.2	10.30	b4 -CH3OH -NH3 [4|5][1-4]	C31H37N3O6
548.2773	548.2755	0.0018	3.2	10.30	z4 -CH3OH [1|2][4-7]	C31H37N3O6
559.3124	559.3126	-0.0002	-0.4	39.71	b5 -C9H10O -NH3 [4|5][1-5]	C29H42N4O7
559.3124	559.3126	-0.0002	-0.4	39.71	z5 -C9H10O [2|3][3-7]	C29H42N4O7
562.2838	562.2912	-0.0073	-13.0	15.55	b4 -H2O -NH3 [4|5][1-4]	C32H39N3O6
562.2838	562.2912	-0.0073	-13.0	15.55	z4 -H2O [1|2][4-7]	C32H39N3O6
580.2996	580.3017	-0.0021	-3.6	43.14	z4 [1|2][4-7]	C32H41N3O7
580.2996	580.3017	-0.0021	-3.6	43.14	b4 -NH3 [4|5][1-4]	C32H41N3O7
625.3120	625.3106	0.0014	2.2	2.12	b5 -C9H11O [3|4][1-5]	C32H42N5O8
642.3566	642.3538	0.0029	4.5	7.04	a4 -H2O -NH3 [1|2][1-4]	C38H47N3O6
642.3566	642.3497	0.0069	10.7	7.04	b5 -C9H10O [7|1][1-5]	C33H47N5O8
661.3545	661.3596	-0.0051	-7.7	13.00	z5 -CH3OH [2|3][3-7]	C37H48N4O7
661.3545	661.3596	-0.0051	-7.7	13.00	a4 [3|4][1-4]	C37H48N4O7
661.3545	661.3596	-0.0051	-7.7	13.00	b5 -CH3OH -NH3 [4|5][1-5]	C37H48N4O7
675.3049	675.2984	0.0065	9.6	2.75	b6 [5|6][1-6]	C31H42N6O11
693.3810	693.3858	-0.0048	-6.9	43.17	b5 -NH3 [4|5][1-5]	C38H52N4O8
693.3810	693.3858	-0.0048	-6.9	43.17	z5 [2|3][3-7]	C38H52N4O8
693.3810	693.3732	0.0078	11.2	43.17	a6 -C9H11O -NH3 [3|4][1-6]	C37H50N5O8
837.4015	837.4029	-0.0014	-1.7	67.46	M -C9H10O -NH3 [7|1][1-7]	C42H56N6O12
854.4250	854.4335	-0.0084	-9.9	81.46	a6 -H2O -NH3 [1|2][1-6]	C47H59N5O10
854.4250	854.4294	-0.0044	-5.2	81.46	M -C9H10O [7|1][1-7]	C42H59N7O12
939.4498	939.4498	-0.0000	-0.0	9.22	M -CH3OH -NH3 [7|1][1-7]	C50H62N6O12
956.4684	956.4764	-0.0080	-8.4	20.80	M -CH3OH [7|1][1-7]	C50H65N7O12
971.4792	971.4761	0.0031	3.2	26.17	M -NH3 [7|1][1-7]	C51H66N6O13

Q-TOF tandem MS

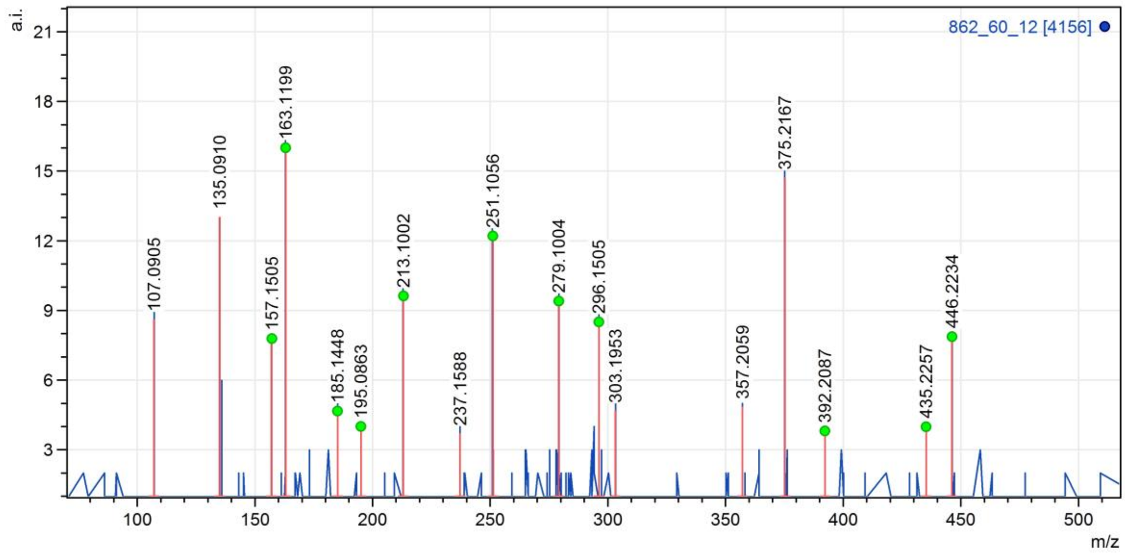

Meas. m/z	Calc. m/z	δ (Da)	δ (ppm)	Rel. Int. (%)	Annotation	Formula
157.1505	157.1335	0.0169	107.9	45.22	a2 [7|1][1-2]	$C_8H_{16}N_2O$
163.1199	163.1117	0.0081	49.9	100.00	z1 -$C_9H_{10}O$ [5|6][7-7]	$C_{11}H_{14}O$
185.1448	185.1285	0.0163	88.1	24.45	b2 [7|1][1-2]	$C_9H_{16}N_2O_2$
195.0863	195.0764	0.0099	50.6	20.00	b2 -H_2O [5|6][1-2]	$C_9H_{10}N_2O_3$
213.1002	213.0870	0.0132	62.2	57.49	b2 [5|6][1-2]	$C_9H_{12}N_2O_4$
251.1056	251.1026	0.0029	11.6	74.72	a2 [2|3][1-2]	$C_{12}H_{14}N_2O_4$
279.1004	279.0975	0.0028	10.1	56.00	b2 [2|3][1-2]	$C_{13}H_{14}N_2O_5$
296.1505	296.1605	-0.0100	-33.7	50.01	b3 +CO [6|7][1-3]	$C_{14}H_{21}N_3O_4$
392.2087	392.2180	-0.0093	-23.6	18.75	b3 -$C_9H_{10}O$ [4|5][1-3]	$C_{20}H_{29}N_3O_5$
435.2257	435.2238	0.0019	4.4	19.99	a4 [7|1][1-4]	$C_{21}H_{30}N_4O_6$
446.2234	446.2286	-0.0051	-11.5	45.83	z4 -$C_9H_{10}O$ [1|2][4-7]	$C_{23}H_{31}N_3O_6$

^{1}H-NMR spectrum of compound 1

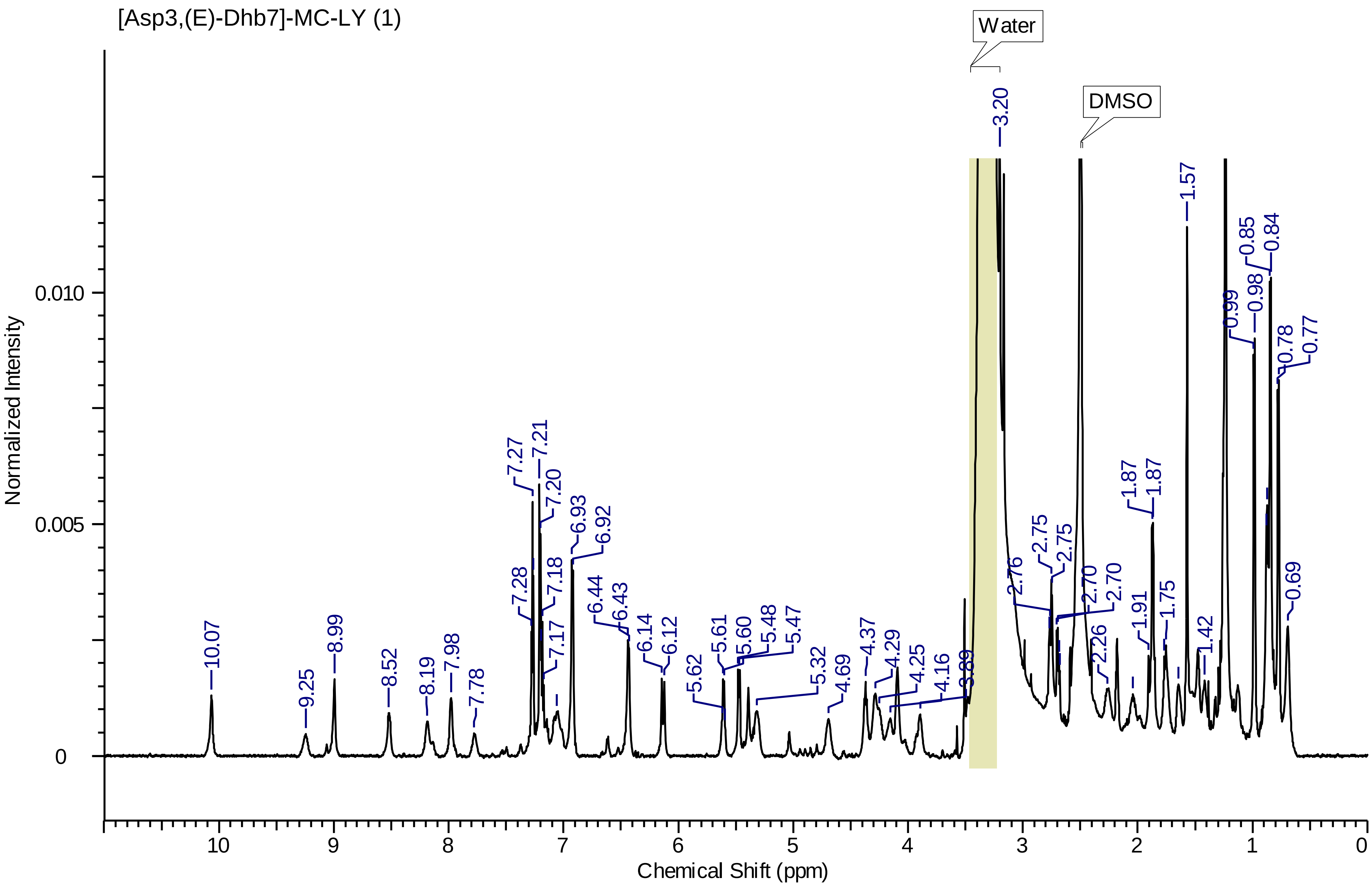

HMQC spectrum of compound 1

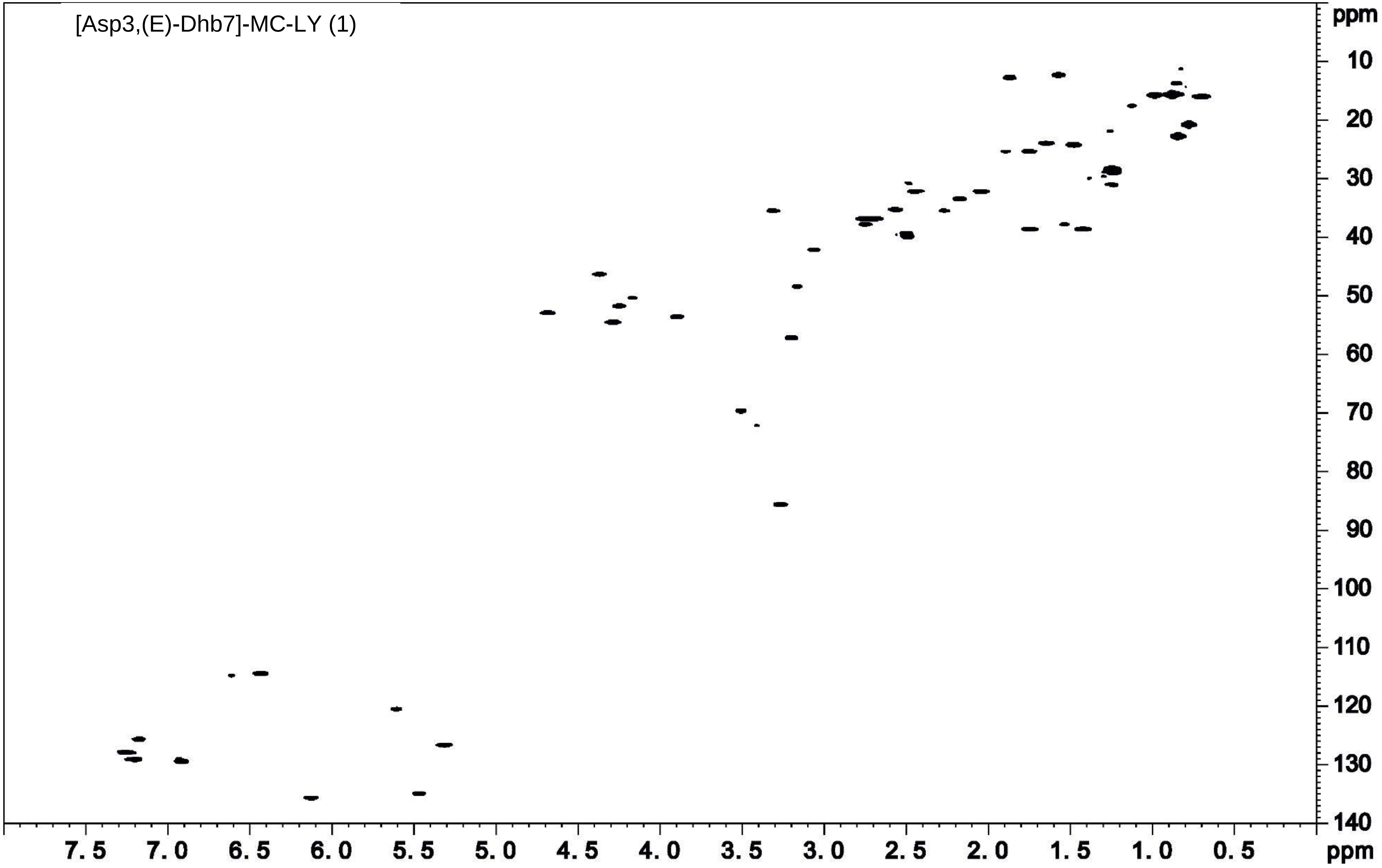

HMBC spectrum of compound 1

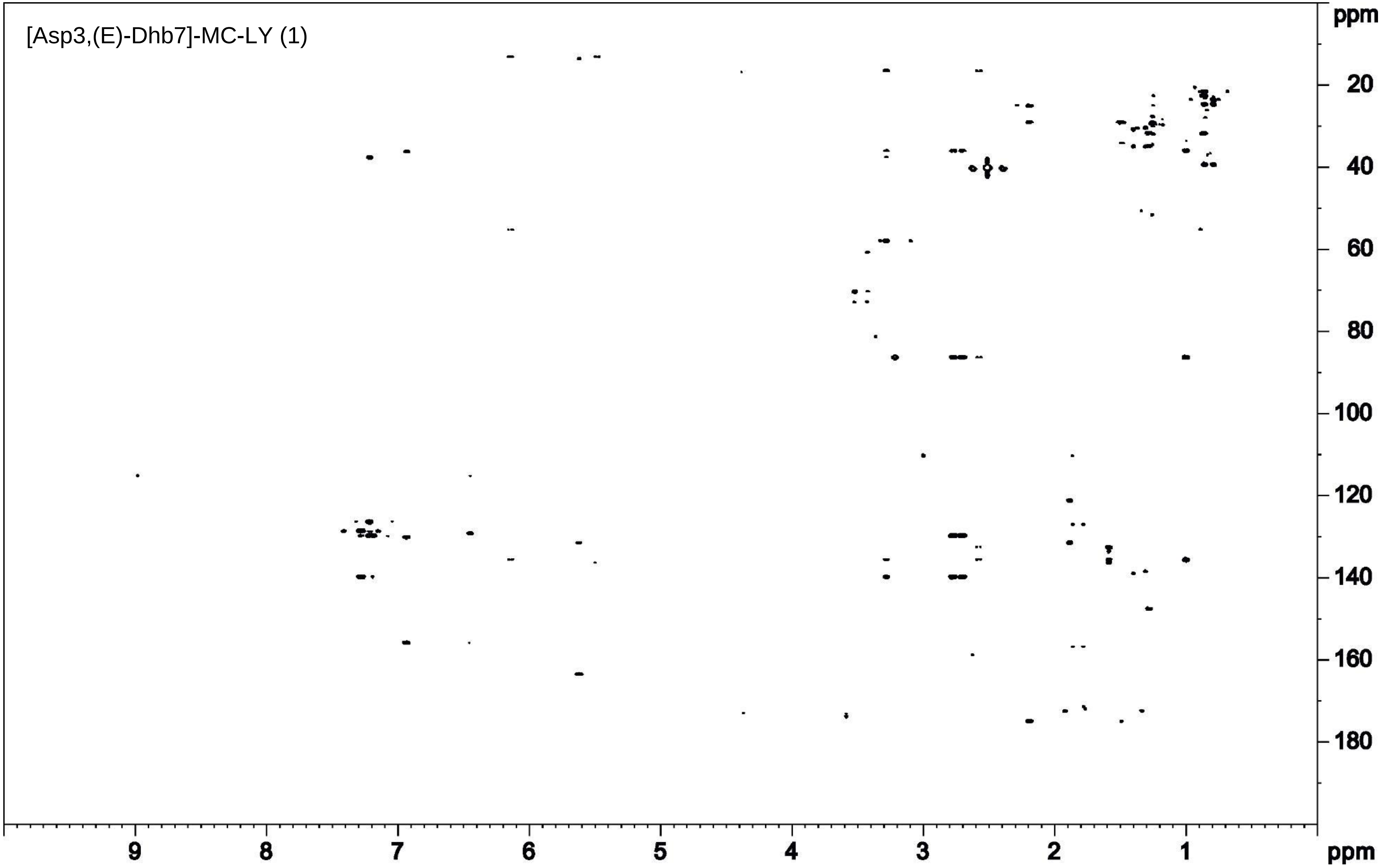

[Asp[3],(*E*)-Dhb[7]]-MC-HtyW (2)

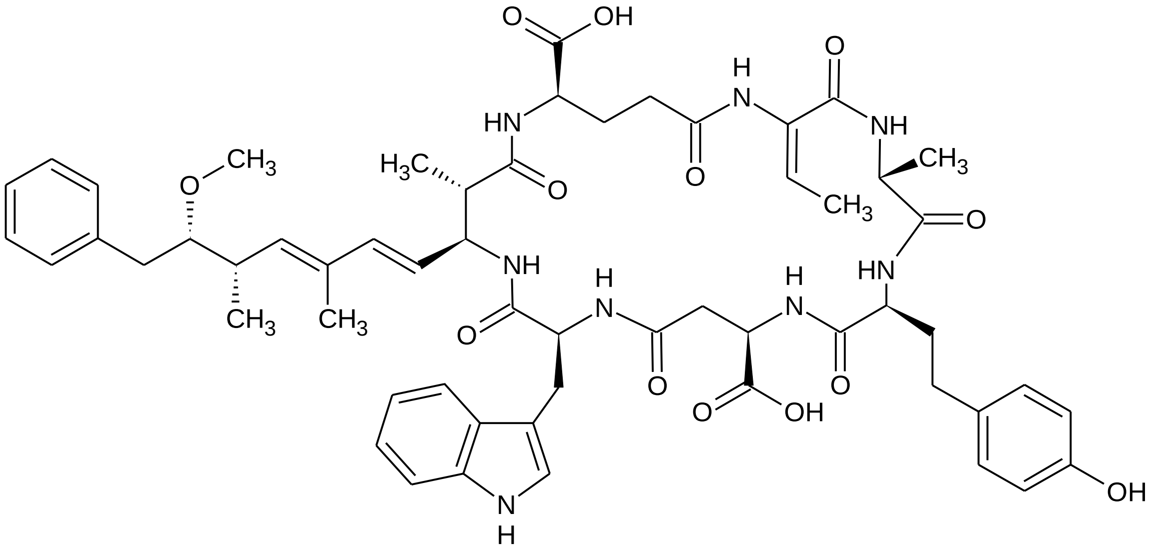

D-Ala | Hty | D-Asp | Trp | Adda | D-Glu | Dhb

Formula (M)	Ion	Meas. m/z	Pred. m/z	Diff (mDa)	Diff (ppm)	Iso Score
C57 H70 N8 O13	[M+H]+	1075.5133	1075.5135 -	0.2 -	0.19	69.04

IT-TOF tandem MS

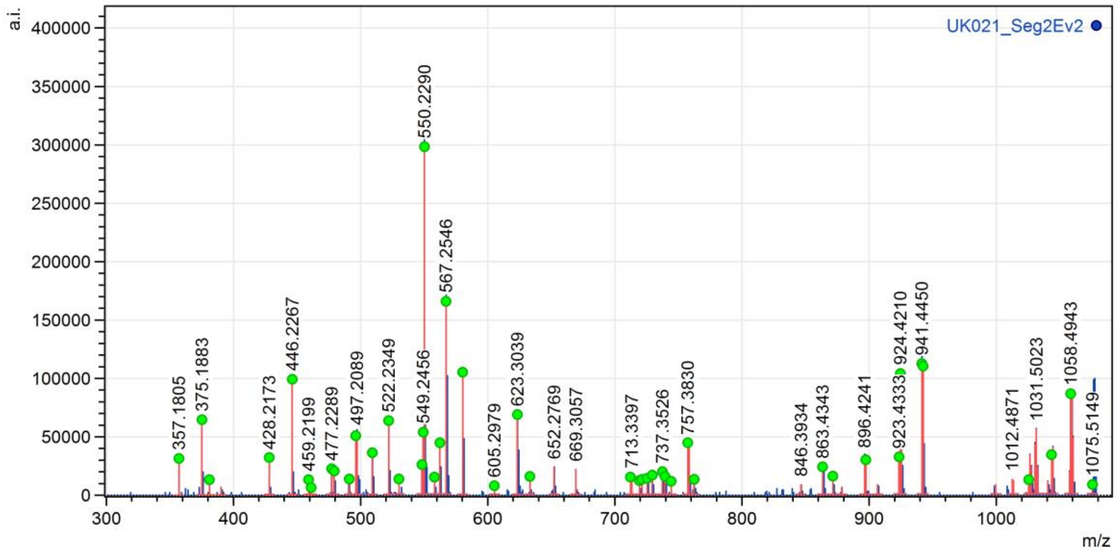

Length	Mo. Mass	Av. Mass	Coverage	Matched Int.
7 (Cyclic)	1074.5062	1075.2140	100.0 %	77.3 %

Meas. m/z	Calc. m/z	δ (Da)	δ (ppm)	Rel. Int. (%)	Annotation	Formula
357.1805	357.1809	-0.0004	-1.1	10.56	z3 -C9H10O -H2O [7|1][5-7]	C20H24N2O4
375.1883	375.1914	-0.0031	-8.4	21.68	z3 -C9H10O [7|1][5-7]	C20H26N2O5
381.1675	381.1769	-0.0094	-24.6	4.41	c3 [7|1][1-3]	C17H24N4O6
428.2173	428.2180	-0.0007	-1.7	10.70	z4 -C9H10O -H2O [1|2][4-7]	C23H29N3O5
446.2267	446.2286	-0.0019	-4.2	33.20	z4 -C9H10O [1|2][4-7]	C23H31N3O6
459.2199	459.2278	-0.0080	-17.3	4.29	z3 -CH3OH -H2O [7|1][5-7]	C28H30N2O4

459.2199	459.2153	0.0046	10.1	4.29	z3 -C9H11O -H2O [6|7][5-7]	C27H28N3O4
461.1987	461.2031	-0.0043	-9.4	1.98	b4 [5|6][1-4]	C22H28N4O7
477.2289	477.2384	-0.0095	-19.8	7.36	b3 -CH3OH -NH3 [4|5][1-3]	C28H32N2O5
477.2289	477.2258	0.0031	6.5	7.36	z3 -C9H11O [6|7][5-7]	C27H30N3O5
479.1925	479.1925	0.0000	0.1	6.73	b3 [1|2][1-3]	C25H26N4O6
491.2515	491.2540	-0.0025	-5.1	4.35	b3 -H2O -NH3 [4|5][1-3]	C29H34N2O5
496.2198	496.2191	0.0007	1.4	16.87	c3 [1|2][1-3]	C25H29N5O6
509.2641	509.2646	-0.0005	-1.0	11.92	b3 -NH3 [4|5][1-3]	C29H36N2O6
522.2349	522.2347	0.0002	0.3	21.13	a4 [7|1][1-4]	C27H31N5O6
530.2561	530.2649	-0.0089	-16.8	4.36	z4 -CH3OH -H2O [1|2][4-7]	C31H35N3O5
548.2772	548.2755	0.0017	3.1	8.45	b4 -CH3OH -NH3 [4|5][1-4]	C31H37N3O6
549.2456	549.2384	0.0072	13.1	17.85	z3 -CH3OH -NH3 [5|6][5-7]	C34H32N2O5
550.2290	550.2296	-0.0007	-1.2	100.00	b4 [7|1][1-4]	C28H31N5O7
558.2212	558.2195	0.0017	3.1	4.88	b5 -H2O [5|6][1-5]	C26H31N5O9
562.2896	562.2912	-0.0016	-2.8	14.80	b4 -H2O -NH3 [4|5][1-4]	C32H39N3O6
567.2546	567.2562	-0.0016	-2.8	55.54	c4 [7|1][1-4]	C28H34N6O7
580.2989	580.3017	-0.0029	-4.9	35.08	b4 -NH3 [4|5][1-4]	C32H41N3O7
605.2979	605.2970	0.0009	1.6	2.46	z5 -C9H10O -H2O [2|3][3-7]	C33H40N4O7
623.3039	623.3075	-0.0036	-5.8	22.97	z5 -C9H10O [2|3][3-7]	C33H42N4O8
633.2742	633.2667	0.0074	11.8	5.12	b5 [6|7][1-5]	C32H36N6O8
712.3425	712.3341	0.0085	11.9	4.93	b4 -CH3OH [2|3][1-4]	C39H45N5O8
712.3425	712.3341	0.0085	11.9	4.93	z5 -C9H10O [5|6][3-7]	C39H45N5O8
719.3325	719.3399	-0.0074	-10.3	3.93	a6 -C9H10O -NH3 [2|3][1-6]	C37H46N6O9
721.3600	721.3596	0.0005	0.7	4.31	z5 -H2O -H2O [2|3][3-7]	C42H48N4O7
725.3582	725.3545	0.0037	5.2	4.60	z5 -CH3OH [2|3][3-7]	C41H48N4O8
729.3588	729.3647	-0.0058	-8.0	5.50	a4 -H2O -NH3 [1|2][1-4]	C44H48N4O6
729.3588	729.3606	-0.0018	-2.5	5.50	b5 -C9H10O [7|1][1-5]	C39H48N6O8
737.3526	737.3505	0.0021	2.9	6.56	b6 -C9H10O -H2O [4|5][1-6]	C37H48N6O10
739.3658	739.3701	-0.0044	-5.9	5.19	b5 -H2O -NH3 [4|5][1-5]	C42H50N4O8
744.3032	744.2988	0.0044	5.9	3.79	b6 -H2O [5|6][1-6]	C37H41N7O10
757.3830	757.3807	0.0023	3.1	14.84	b5 -NH3 [4|5][1-5]	C42H52N4O9
762.3153	762.3093	0.0059	7.8	4.32	b6 [5|6][1-6]	C37H43N7O11
863.4343	863.4338	0.0005	0.6	7.89	b5 [7|1][1-5]	C48H58N6O9
871.4274	871.4236	0.0038	4.4	5.26	b6 -H2O [4|5][1-6]	C46H58N6O11
897.4226	897.4182	0.0045	5.0	9.88	b6 -CH3OH -NH3 [6|7][1-6]	C51H56N6O9
923.4333	923.4298	0.0035	3.8	10.67	M -C9H10O -H2O [7|1][1-7]	C48H58N8O11
924.4210	924.4138	0.0073	7.8	34.76	M -C9H10O -NH3 [7|1][1-7]	C48H57N7O12
925.4215	925.4131	0.0085	9.1	31.95	z6 -CH3OH -H2O [6|7][2-7]	C52H56N6O10
941.4450	941.4403	0.0046	4.9	37.54	M -C9H10O [7|1][1-7]	C48H60N8O12
942.4466	942.4396	0.0069	7.4	36.78	b6 +CO -CH3OH [6|7][1-6]	C52H59N7O10
942.4466	942.4396	0.0069	7.4	36.78	b6 -CH3OH -H2O [7|1][1-6]	C52H59N7O10
1025.4828	1025.4767	0.0061	5.9	4.03	M -CH3OH -H2O [7|1][1-7]	C56H64N8O11
1043.4912	1043.4873	0.0039	3.7	11.25	M -CH3OH [7|1][1-7]	C56H66N8O12
1058.4943	1058.4870	0.0074	7.0	28.80	M -NH3 [7|1][1-7]	C57H67N7O13

Q-TOF tandem MS

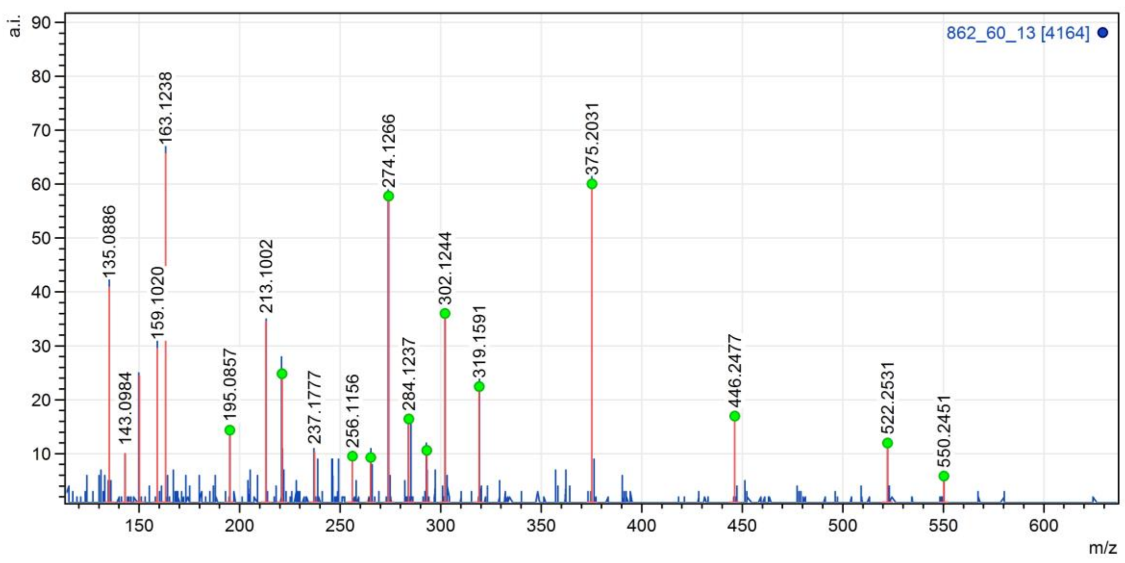

Meas. m/z	Calc. m/z	δ (Da)	δ (ppm)	Rel.Int. (%)	Annotation	Formula
195.0857	195.0764	0.0093	47.7	20.68	b2 -H2O [5|6][1-2]	C9H10N2O3
221.1297	221.1285	0.0012	5.5	36.87	a2 [7|1][1-2]	C12H16N2O2
256.1156	256.1081	0.0075	29.4	13.21	a2 -H2O [2|3][1-2]	C14H13N3O2
265.1675	265.1587	0.0088	33.3	12.90	z1 -CH3OH [5|6][7-7]	C19H20O
274.1266	274.1186	0.0080	29.3	87.86	a2 [2|3][1-2]	C14H15N3O3
284.1237	284.1241	-0.0004	-1.3	23.88	b3 [5|6][1-3]	C12H17N3O5
293.1310	293.1244	0.0066	22.5	14.93	c3 +CO -H2O -H2O [5|6][1-3]	C13H16N4O4
302.1244	302.1135	0.0109	35.9	54.17	b2 [2|3][1-2]	C15H15N3O4
319.1591	319.1652	-0.0062	-19.3	33.22	b2+CO-C9H10O -H2O [4|5][1-2]	C17H22N2O4
375.2031	375.1914	0.0116	31.0	91.43	z3 -C9H10O [7|1][5-7]	C20H26N2O5
446.2477	446.2286	0.0192	42.9	24.77	b4 -C9H10O -NH3 [4|5][1-4]	C23H31N3O6
522.2531	522.2347	0.0184	35.2	17.03	a4 [7|1][1-4]	C27H31N5O6
550.2451	550.2296	0.0154	28.1	7.50	b4 [7|1][1-4]	C28H31N5O7

^{1}H-NMR spectrum of compound 2

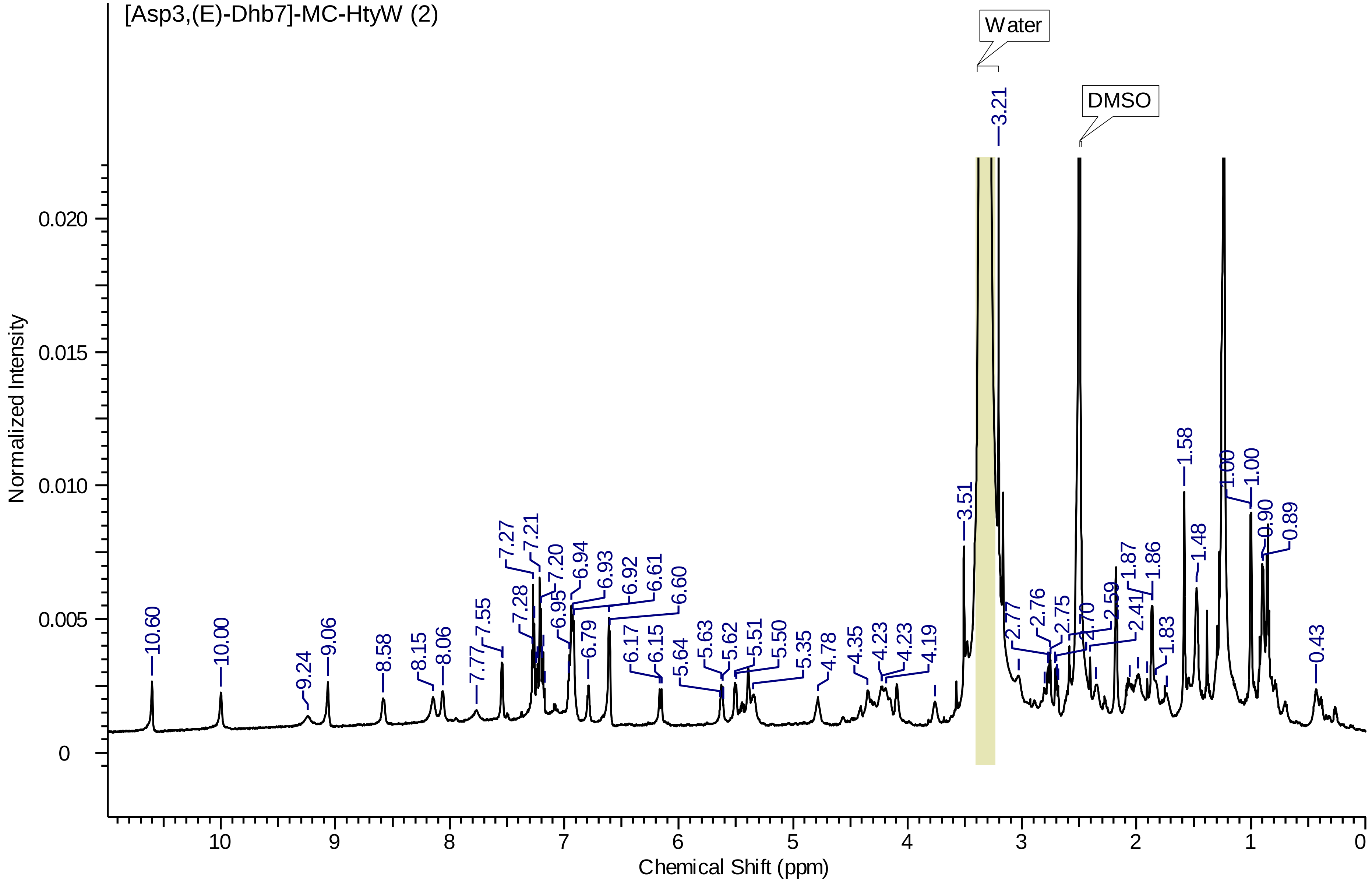

HMQC spectrum of compound 2

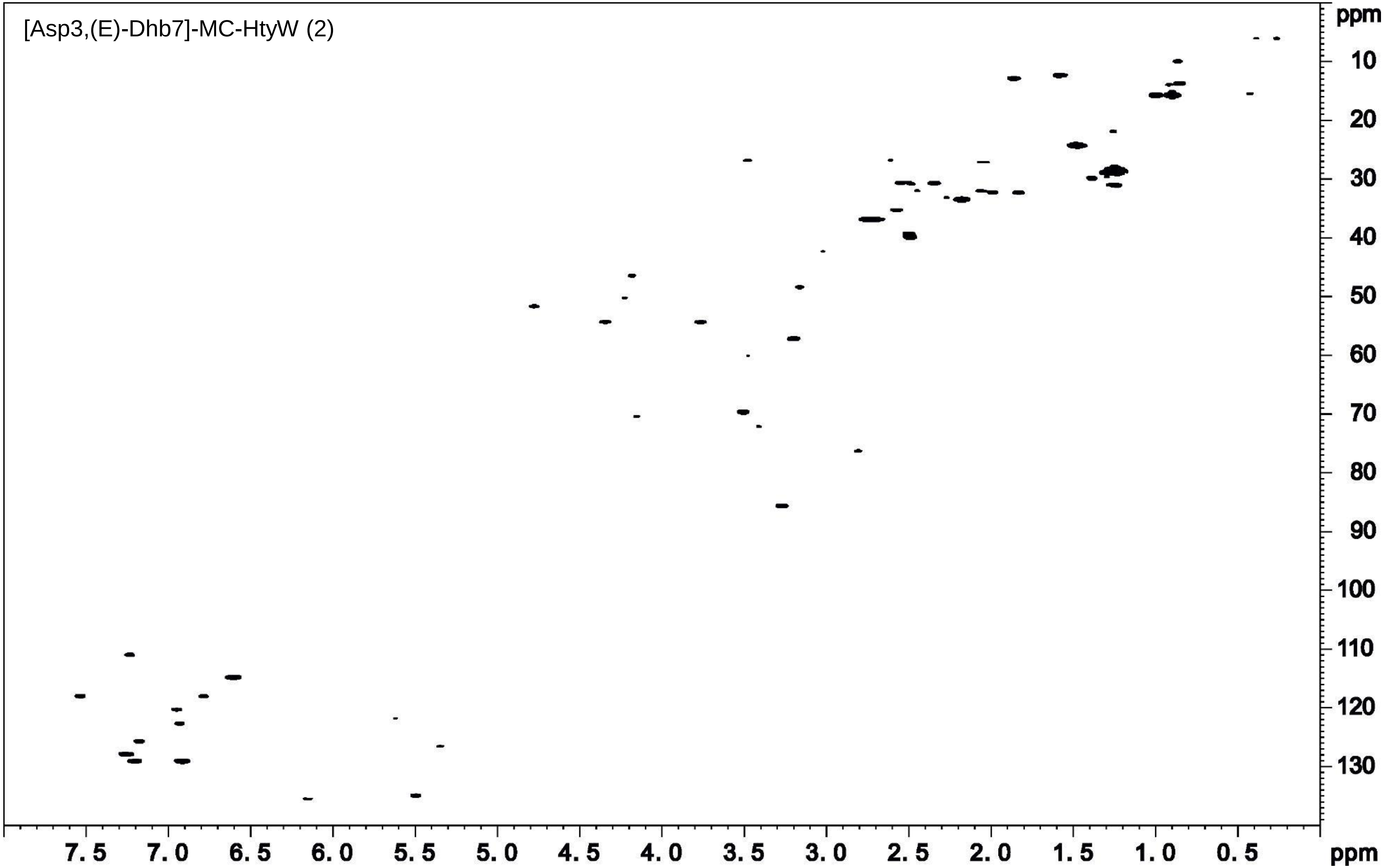

HMBC spectrum of compound 2

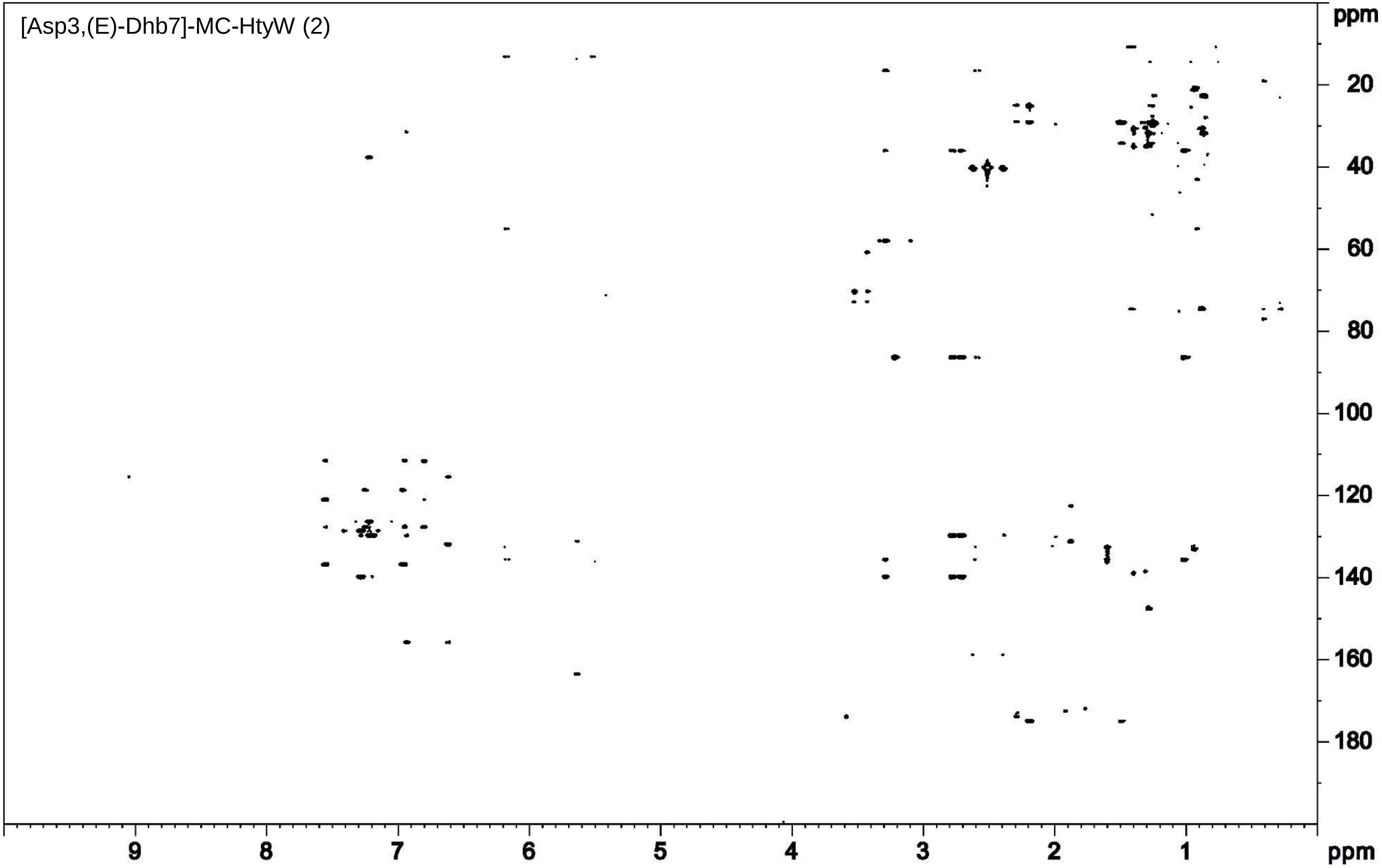

[Asp³,(*E*)-Dhb⁷]-MC-LW (3)

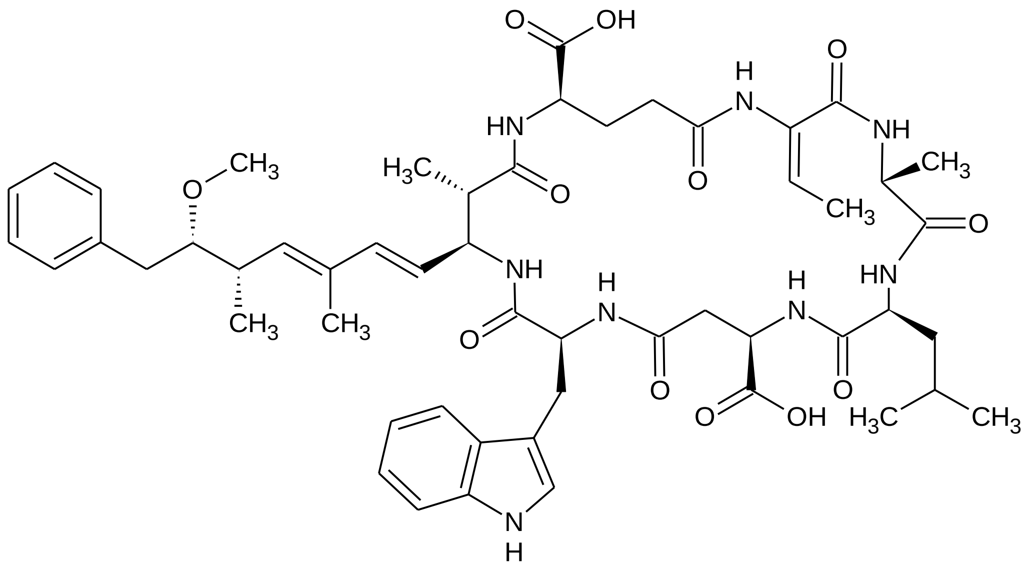

D-Ala | Leu | D-Asp | Trp | Adda | D-Glu | Dhb

Formula (M)	Ion	Meas. m/z	Pred. m/z	Diff (mDa)	Diff (ppm)	Iso Score
C53 H70 N8 O12	[M+H]+	1011.5172	1011.5186	-1.4	-1.38	79.91

IT-TOF tandem MS

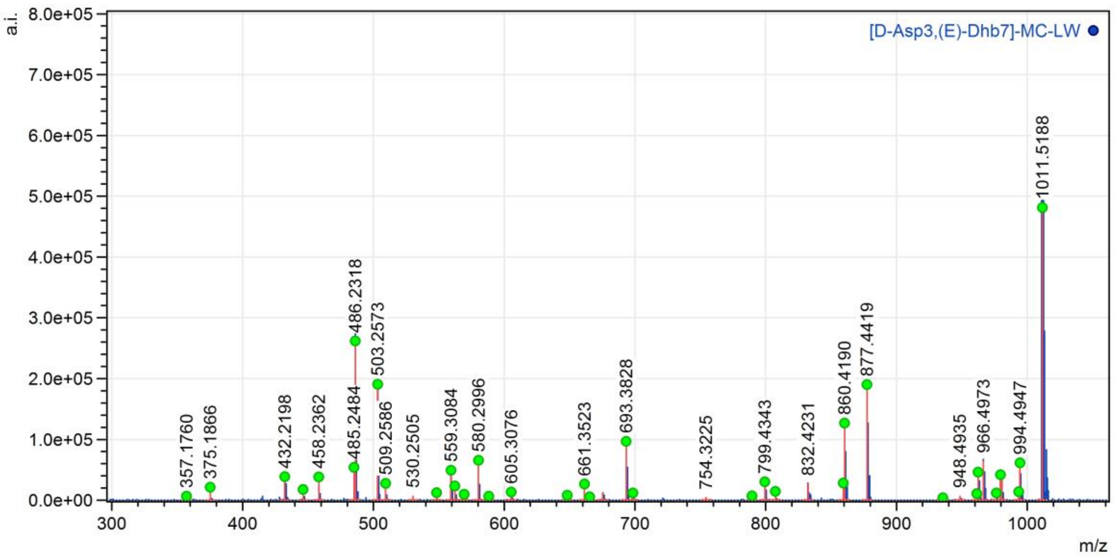

Length	Mo. Mass	Av. Mass	Coverage	Matched Int.
7 (Cyclic)	1010.5113	1011.1717	100.0 %	90.4 %

Meas. m/z	Calc. m/z	δ (Da)	δ (ppm)	Rel. Int. (%)	Annotation	Formula
357.1760	357.1809	-0.0048	-13.5	1.35	z3 -C9H10O -H2O [7|1][5-7]	C20H24N2O4
375.1866	375.1914	-0.0049	-13.0	4.42	z3 -C9H10O [7|1][5-7]	C20H26N2O5
432.2198	432.2241	-0.0044	-10.1	8.02	c3 [1|2][1-3]	C21H29N5O5
446.2263	446.2286	-0.0022	-5.0	3.63	z4 -C9H10O [1|2][4-7]	C23H31N3O6
458.2362	458.2398	-0.0036	-7.8	7.94	a4 [7|1][1-4]	C23H31N5O5
486.2318	486.2347	-0.0029	-5.9	54.38	b4 [7|1][1-4]	C24H31N5O6
503.2573	503.2613	-0.0040	-7.9	39.55	c4 [7|1][1-4]	C24H34N6O6

509.2586	509.2646	-0.0060	-11.7	5.64	z3 [7|1][5-7]	C29H36N2O6
509.2586	509.2646	-0.0060	-11.7	5.64	b3 -NH3 [4|5][1-3]	C29H36N2O6
548.2727	548.2755	-0.0029	-5.2	2.52	z4 -CH3OH [1|2][4-7]	C31H37N3O6
548.2727	548.2755	-0.0029	-5.2	2.52	b4 -CH3OH -NH3 [4|5][1-4]	C31H37N3O6
559.3084	559.3126	-0.0043	-7.6	10.17	b5 -C9H10O -NH3 [4|5][1-5]	C29H42N4O7
559.3084	559.3126	-0.0043	-7.6	10.17	z5 -C9H10O [2|3][3-7]	C29H42N4O7
562.2890	562.2912	-0.0022	-3.9	4.76	b4 -H2O -NH3 [4|5][1-4]	C32H39N3O6
562.2890	562.2912	-0.0022	-3.9	4.76	z4 -H2O [1|2][4-7]	C32H39N3O6
569.2633	569.2718	-0.0085	-15.0	1.96	b5 [6|7][1-5]	C28H36N6O7
580.2996	580.3017	-0.0022	-3.7	13.54	b4 -NH3 [4|5][1-4]	C32H41N3O7
580.2996	580.3017	-0.0022	-3.7	13.54	z4 [1|2][4-7]	C32H41N3O7
588.2876	588.2817	0.0059	10.0	1.29	b4 +CO -C9H10O -H2O [3|4][1-4]	C32H37N5O6
605.3076	605.3082	-0.0006	-0.9	2.71	c4 +CO -C9H10O -H2O [3|4][1-4]	C32H40N6O6
648.3367	648.3392	-0.0025	-3.9	1.60	b5 -C9H10O -NH3 [7|1][1-5]	C35H45N5O7
648.3367	648.3392	-0.0025	-3.9	1.60	z5 -C9H10O [5|6][3-7]	C35H45N5O7
661.3523	661.3596	-0.0073	-11.0	5.42	b5 -CH3OH -NH3 [4|5][1-5]	C37H48N4O7
661.3523	661.3596	-0.0073	-11.0	5.42	z5 -CH3OH [2|3][3-7]	C37H48N4O7
665.3734	665.3697	0.0037	5.6	1.03	a4 -H2O -NH3 [1|2][1-4]	C40H48N4O5
665.3734	665.3657	0.0077	11.6	1.03	b5 -C9H10O [7|1][1-5]	C35H48N6O7
693.3828	693.3858	-0.0030	-4.3	20.07	z5 [2|3][3-7]	C38H52N4O8
693.3828	693.3858	-0.0030	-4.3	20.07	b5 -NH3 [4|5][1-5]	C38H52N4O8
698.3109	698.3144	-0.0035	-5.1	2.34	b6 [5|6][1-6]	C33H43N7O10
789.4081	789.4056	0.0025	3.2	1.36	b6 +CO -C9H11O [3|4][1-6]	C41H54N7O9
799.4343	799.4389	-0.0046	-5.8	6.15	b5 [7|1][1-5]	C44H58N6O8
807.4200	807.4287	-0.0087	-10.8	2.86	b6 -H2O [4|5][1-6]	C42H58N6O10
859.4343	859.4349	-0.0006	-0.7	5.71	M -C9H10O -H2O [7|1][1-7]	C44H58N8O10
860.4190	860.4189	0.0001	0.1	26.26	M -C9H10O -NH3 [7|1][1-7]	C44H57N7O11
877.4419	877.4495	-0.0076	-8.7	39.41	a6 -H2O -NH3 [1|2][1-6]	C49H60N6O9
877.4419	877.4454	-0.0036	-4.1	39.41	M -C9H10O [7|1][1-7]	C44H60N8O11
935.4651	935.4662	-0.0010	-1.1	0.71	c6 +CO -CH3OH -H2O [1|2][1-6]	C50H62N8O10
961.4832	961.4818	0.0014	1.4	2.09	M -CH3OH -H2O [7|1][1-7]	C52H64N8O10
962.4716	962.4658	0.0058	6.0	9.51	M -CH3OH -NH3 [7|1][1-7]	C52H63N7O11
976.4822	976.4815	0.0007	0.7	2.34	M -H2O -NH3 [7|1][1-7]	C53H65N7O11
979.4930	979.4924	0.0006	0.6	8.60	M -CH3OH [7|1][1-7]	C52H66N8O11
993.5082	993.5080	0.0002	0.2	2.82	M -H2O [7|1][1-7]	C53H68N8O11
994.4947	994.4920	0.0026	2.6	12.67	M -NH3 [7|1][1-7]	C53H67N7O12
1011.5188	1011.5186	0.0002	0.2	100.00	M [7|1][1-7]	C53H70N8O12

Q-TOF tandem MS

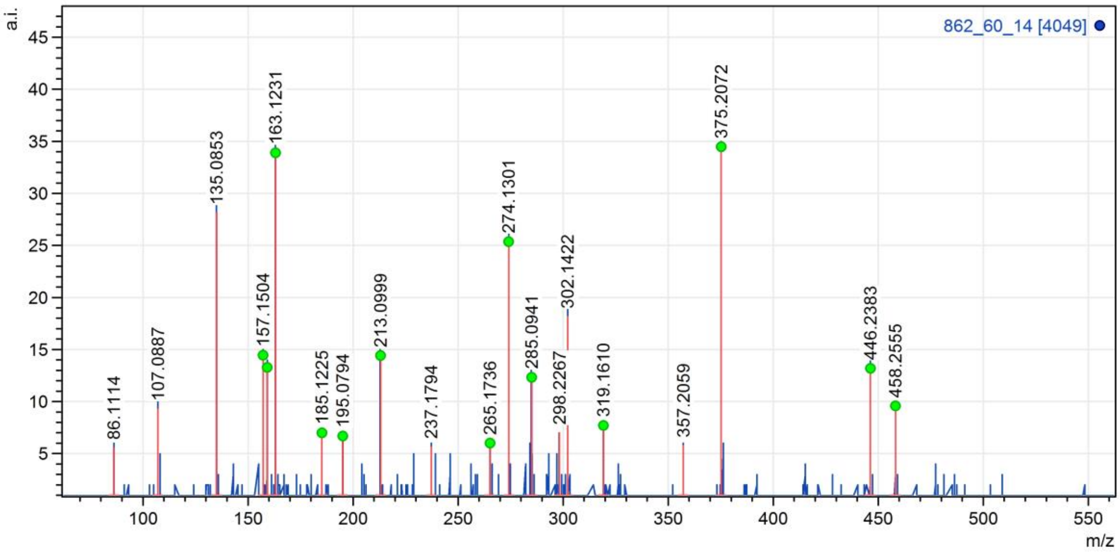

Meas. m/z	Calc. m/z	δ (Da)	δ (ppm)	Rel. Int. (%)	Annotation	Formula
157.1504	157.1335	0.0169	107.4	40.23	a2 [7|1][1-2]	C8H16N2O
159.0997	159.1128	-0.0131	-82.2	36.68	c1 +CO [1|2][1-1]	C7H14N2O2
163.1231	163.1117	0.0114	69.8	98.33	z1 -C9H10O [5|6][7-7]	C11H14O
185.1225	185.1285	-0.0059	-32.0	17.92	b2 [7|1][1-2]	C9H16N2O2
195.0794	195.0764	0.0030	15.5	17.03	b2 -H2O [5|6][1-2]	C9H10N2O3
213.0999	213.0870	0.0129	60.6	40.08	b2 [5|6][1-2]	C9H12N2O4
265.1736	265.1587	0.0149	56.2	14.94	z1 -CH3OH [5|6][7-7]	C19H20O
274.1301	274.1438	-0.0137	-49.9	72.83	z2 -C9H10O -H2O [6|7][6-7]	C16H19NO3
274.1301	274.1186	0.0115	41.9	72.83	a2 [2|3][1-2]	C14H15N3O3
285.0941	285.0870	0.0072	25.1	33.86	b2 -NH3 [2|3][1-2]	C15H12N2O4
319.1610	319.1765	-0.0155	-48.5	20.04	a4 -H2O -H2O [6|7][1-4]	C16H22N4O3
375.2072	375.1914	0.0157	42.0	100.00	z3 -C9H10O [7|1][5-7]	C20H26N2O5
446.2383	446.2286	0.0097	21.8	36.45	b4 -C9H10O -NH3 [4|5][1-4]	C23H31N3O6
458.2555	458.2398	0.0157	34.2	25.62	a4 [7|1][1-4]	C23H31N5O5

^{1}H-NMR spectrum of compound 3

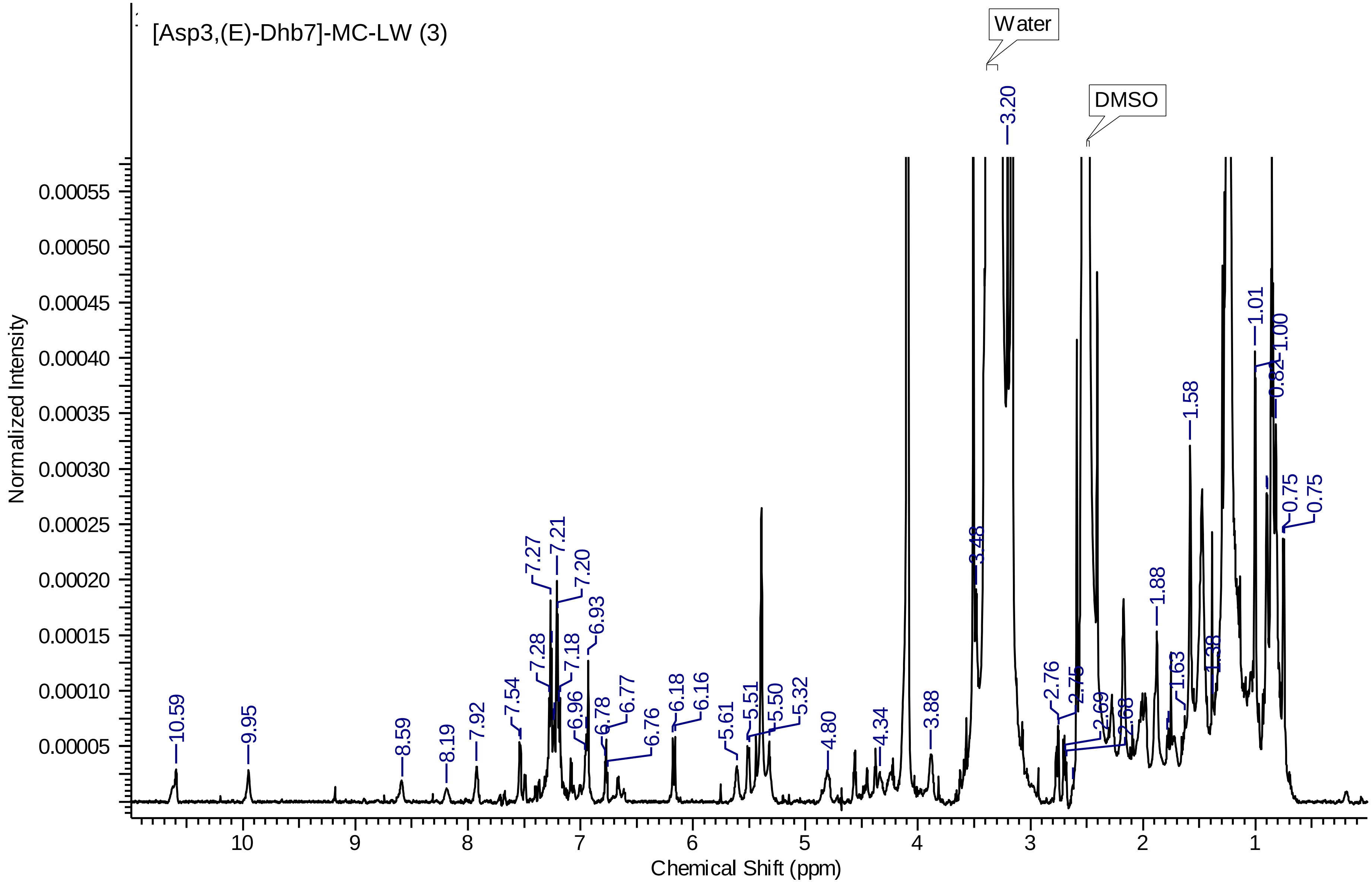

Python Script for MC congener calculation and its output

Script

```
import itertools

monomers = [
#Position 1: only Ala known in MCs from Planktothrix or Oscillatoria
  [71.0371],
#Position 2: make all known monomers in this flexible position possible; do not apply restrictions indicated by biosynthesis genes
  [113.0841, 71.0371, 163.0633, 129.0426, 99.0684, 143.0582, 156.1011, 147.0684, 147.0354, 161.0841, 177.0790, 186.0793, 170.1168, 127.0997, 167.0946],
#Position 3: unmethylated Asp highly likely
  [115.0269],
#Position 4: make all known monomers in this flexible position possible; do not apply restrictions indicated by biosynthesis genes
  [71.0371, 85.0528, 113.0841, 156.1011, 129.0426, 143.0582, 147.0684, 163.0633, 186.0793, 147.0354, 170.1168, 177.0790, 161.0841, 85.0528],
#Position 5: only Adda known in MCs from Planktothrix or Oscillatoria
  [313.2042],
#Position 6: Glu highly likely
  [129.0426],
#Position 7: strain can only synthesize Dhb
  [83.0371],
]

monomers = [item for item in monomers if item != []]

combinations = list(itertools.product(*monomers))

sums = [sum(item) for item in combinations]

both = sorted(zip(combinations, sums), key=lambda both: both[1])

f = open('output.csv','wb')
f.write('Molecular Mass' + ';' + ';'.join('Monomer ' + str(i+1) for i in range(len(monomers))) + '\r\n')

for item in both:
    f.write(str(item[1]) + ';' + ';'.join([str(x) for x in item[0]]) + '\r\n')

f.close()
```

Output

Manually filtered for observed molecular weights around 987.497, 1010.509, and 1074.505
Indicated in bold: final established structure after evaluation of NMR and tandem MS data

Molecular Mass	Monomer 1	Monomer 2	Monomer 3	Monomer 4	Monomer 5	Monomer 6	Monomer 7
**987.4953**	**71.0371**	**113.0841**	**115.0269**	**163.0633**	**313.2042**	**129.0426**	**83.0371**
987.4953	71.0371	163.0633	115.0269	113.0841	313.2042	129.0426	83.0371
987.4953	71.0371	99.0684	115.0269	177.079	313.2042	129.0426	83.0371
1010.5072	71.0371	143.0582	115.0269	156.1011	313.2042	129.0426	83.0371
1010.5072	71.0371	156.1011	115.0269	143.0582	313.2042	129.0426	83.0371
1010.5073	71.0371	129.0426	115.0269	170.1168	313.2042	129.0426	83.0371
1010.5073	71.0371	170.1168	115.0269	129.0426	313.2042	129.0426	83.0371
**1010.5113**	**71.0371**	**113.0841**	**115.0269**	**186.0793**	**313.2042**	**129.0426**	**83.0371**
1010.5113	71.0371	186.0793	115.0269	113.0841	313.2042	129.0426	83.0371
**1074.5062**	**71.0371**	**177.079**	**115.0269**	**186.0793**	**313.2042**	**129.0426**	**83.0371**
1074.5062	71.0371	186.0793	115.0269	177.079	313.2042	129.0426	83.0371

EC_{50} calculation

The script used for the calculation of EC_{50} values can be found below, followed by the graphical output of the script.
Currently, a Graphical User Interface (GUI) for the tool $EC_{50}^{calculator}$ is under development. This, as well as the validation of the tool performance, will be reported separately.

Script

```
# -*- coding: utf-8 -*-

"""
This script is the backbone of the software EC50calculator. A GUI for this
tool is currently under development.

This script is licensed CC-BY.
You are free:
    to Share: to copy, distribute and transmit the work
    to Remix: to adapt the work
    to make commercial use of the work
Under the following conditions:
    Attribution: You must attribute the work in the manner specified by the
    author or licensor (but not in any way that suggests that they endorse
    you or your use of the work).
For more information see http://creativecommons.org/licenses/by/2.0/
"""

from itertools import cycle
import numpy as np
import matplotlib.pyplot as plt
from scipy.optimize import curve_fit

# Settings.
BLACK_AND_WHITE = False
DRAW_ERROR_BARS = True
X_UNIT = 'nM'

def four_parameter_logistic_model(x, ec50, slope, top, bottom):
    """Calculate estimates using 4PL equation."""
    return bottom + ((top - bottom) / (1 + (x/ec50)**slope))

# Compounds data.
compounds = []

# Input of experimental X and Y data as well as Y-error.
compounds.append({
    'name': 'MC-LR',
    'xdata': np.array([0.03943662, 0.078873239, 0.394366197, 0.788732394,
                       3.943661972, 7.887323944, 39.43661972, 78.87323944,
                       394.3661972, 788.7323944]),
    'ydata': np.array([89.84341938, 91.67904903, 83.11468472, 83.58107501,
                       15.61802767, 14.17689378, 6.389301634, 5.924672027,
                       0.29717682, 0.891530461]),
    'sd': np.array([3.746359606, 0.251566602, 3.560375452, 3.640321726,
                    0.554277319, 1.159312281, 0.424481267, 1.59888909,
                    0.440853711, 1.29560105])
})
```

```python
compounds.append({
    'name': 'MC-HtyY',
    'xdata': np.array([0.023453853, 0.046907707, 0.234538535, 0.469077069,
                       2.345385347, 4.690770695, 23.45385347, 46.90770695,
                       234.5385347, 469.0770695]),
    'ydata': np.array([93.22560597, 98.63269111, 81.64556962, 92.66244168,
                       59.30535123, 33.37476694, 13.98384089, 12.65822785,
                       5.497771174, 5.221518987]),
    'sd': np.array([1.156027235, 1.701948577, 4.42834711, 4.29218852,
                    4.302243708, 4.005304042, 1.674676021, 1.180642196,
                    0.938254754, 1.073210344])
})

compounds.append({
    'name': 'MC-HtyHty',
    'xdata': np.array([0.008591549, 0.017183099, 0.085915493, 0.171830986,
                       0.85915493, 1.718309859, 8.591549296, 17.18309859,
                       85.91549296, 171.8309859]),
    'ydata': np.array([104.0365985, 109.6878364, 107.7502691, 103.0678149,
                       87.56727664, 80.13662638, 25.90646348, 17.10769231,
                       10.38751346, 6.516027325]),
    'sd': np.array([7.124556368, 4.489801528, 6.301014592, 7.723351793,
                    5.62632281, 4.926057796, 2.142121388, 0.989825707,
                    1.424439145, 2.785592448])
})

compounds.append({
    'name': 'MC-LY (1)',
    'xdata': np.array([0.014463019, 0.028926039, 0.144630193, 0.289260385,
                       1.446301925, 2.89260385, 14.46301925, 28.9260385,
                       144.6301925, 289.260385]),
    'ydata': np.array([95.53770638, 90.98101266, 95.25316456, 94.77848101,
                       68.98734177, 56.80379747, 22.57920571, 17.7599286,
                       6.962025316, 4.574811625]),
    'sd': np.array([8.118161627, 7.962248067, 10.8621385, 6.703991743,
                    19.08080434, 8.838711702, 3.927820396, 1.26252992,
                    2.004821903, 0.834538341])
})

compounds.append({
    'name': 'MC-HtyW (2)',
    'xdata': np.array([0.010335196, 0.020670391, 0.103351955, 0.206703911,
                       1.033519553, 2.067039106, 10.33519553, 20.67039106,
                       103.3519553, 206.7039106]),
    'ydata': np.array([95.2765693, 98.07333748, 97.32753263, 100.8073197,
                       84.83530143, 77.56370416, 32.8154133, 23.62755651,
                       10.06836544, 9.322560597]),
    'sd': np.array([8.889146668, 9.404856199, 9.660657541, 3.165519847,
                    8.747664581, 11.39295451, 5.176340653, 1.416905335,
                    1.251707784, 0.326178133])
})

compounds.append({
    'name': 'MC-LW (3)',
    'xdata': np.array([0.002376238, 0.004752475, 0.023762376, 0.047524752,
                       0.237623762, 0.475247525, 2.376237624, 4.752475248,
                       23.76237624, 47.52475248]),
    'ydata': np.array([90.18987342, 91.36109385, 96.95463021, 95.88607595,
                       89.24050633, 90.49034175, 74.84177215, 56.86761964,
                       22.00124301, 17.24683544]),
    'sd': np.array([2.446214043, 2.645124482, 1.378518003, 3.663303034,
                    3.317751097, 4.351216653, 3.418672395, 5.251377734,
                    1.552341365, 1.698100488])
})
```

```
for compound in compounds:

    # Fit curve.
    popt, pcov, infodict, errmsg, ier = curve_fit(
        four_parameter_logistic_model,
        compound['xdata'],
        compound['ydata'],
        full_output=True)

    # Calculate additional parameters and write all
    # calculated parameters in compound dictionary.
    compound['ec50'] = popt[0]
    compound['slope'] = popt[1]
    compound['top'] = popt[2]
    compound['bottom'] = popt[3]
    compound['sd_ec50'] = pcov[0, 0]**0.5
    compound['sd_slope'] = pcov[1, 1]**0.5
    compound['percent_fitting_error'] = (pcov[0, 0]**0.5) / popt[0] * 100

    # Calculate R squared.
    sum_of_squares = (infodict['fvec']**2).sum()
    ss_tot = ((compound['ydata']-compound['ydata'].mean())**2).sum()
    rsquared = 1-(sum_of_squares/ss_tot)

    compound['sum_of_squares'] = sum_of_squares
    compound['rsquared'] = rsquared

    # Calculate points of fitted curve and put them in the
    # compound dictionary.
    x = np.linspace(
        (compound['xdata'].min() - 0.5 * compound['xdata'].min()),
        (compound['xdata'].max() + 0.5 * compound['xdata'].max()),
        ((compound['xdata'].max() - compound['xdata'].min()) * 50)
    )
    y = four_parameter_logistic_model(x, *popt)
    compound['x'] = x
    compound['y'] = y

    print 60*'-'
    print compound['name']
    print 'EC50: %s' % compound['ec50']
    print 'Slope: %s' % compound['slope']
    print 'Top asymptote: %s' %compound['top']
    print 'Bottom asymptote: %s' % compound['bottom']
    print 'SD EC50: %s' % compound['sd_ec50']
    print 'SD Slope %s' % compound['sd_slope']
    print '%% Fitting Error: %s' % compound['percent_fitting_error']
    print 'Sum of squares: %s' % compound['sum_of_squares']
    print 'R squared: %s' % compound['rsquared']


# Create and show plot for all compounds.

# Define markers and colors for different plot styles:
# "color" or "black & white"
markers = cycle(['s', '^', 'v', 'd', 'o', '*'])
colors = cycle(['b', 'g', 'r', 'c', 'm', 'k'])
lines = cycle(['-', '--', '-.', ':', '.'])
```

```python
# Calculate x- and y-axis parameters that fit to
# plot all curves in one graph.
xmax = np.max([compound['xdata'] for compound in compounds])
xmin = np.min([compound['xdata'] for compound in compounds])
if compound['sd'] is not None:
    ymax = np.max([(compound['ydata'] + compound['sd']) for
                   compound in compounds])
    ymin = np.min([(compound['ydata'] - compound['sd']) for
                   compound in compounds])
else:
    ymax = np.max([compound['ydata'] for compound in compounds])
    ymin = np.min([compound['ydata'] for compound in compounds])

for compound in compounds:

    # Define marker/line style/color depending on choice
    # "color" or "black & white"
    if BLACK_AND_WHITE:
        marker = markers.next()
        color = 'k'
        linestyle = lines.next()
    else:
        marker = 'o'
        color = colors.next()
        linestyle = '-'

    plt.semilogx(compound['xdata'],
                 compound['ydata'],
                 linestyle='None',
                 marker=marker,
                 color=color,
                 markeredgecolor='none',
                 label=compound['name'])
    plt.semilogx(compound['x'],
                 compound['y'],
                 linestyle=linestyle,
                 label='fit %s' % compound['name'],
                 color=color)
    if compound['sd'] is not None and DRAW_ERROR_BARS is True:
        plt.errorbar(compound['xdata'],
                     compound['ydata'],
                     yerr=compound['sd'],
                     ecolor='k',
                     fmt=None)

# Adjust x- and y-axes according to data.
plt.ylim(np.floor(ymin/10)*10, np.ceil(ymax/10)*10)
plt.xlim(xmin*0.2, xmax*8)

# Adjust ticks according to y-axis range.
plt.yticks(range(int(np.floor(ymin/10)*10),
           int(np.ceil(ymax/10)*10+10), 10))

# Decorate graph.
plt.title('Dose-Response-Curves')
plt.xlabel('Concentration [%s]' % X_UNIT)
plt.ylabel('Activity [%]')
plt.legend(loc='upper right', numpoints=1, prop={'size': 10})
plt.grid(True)


plt.show()
```

Output

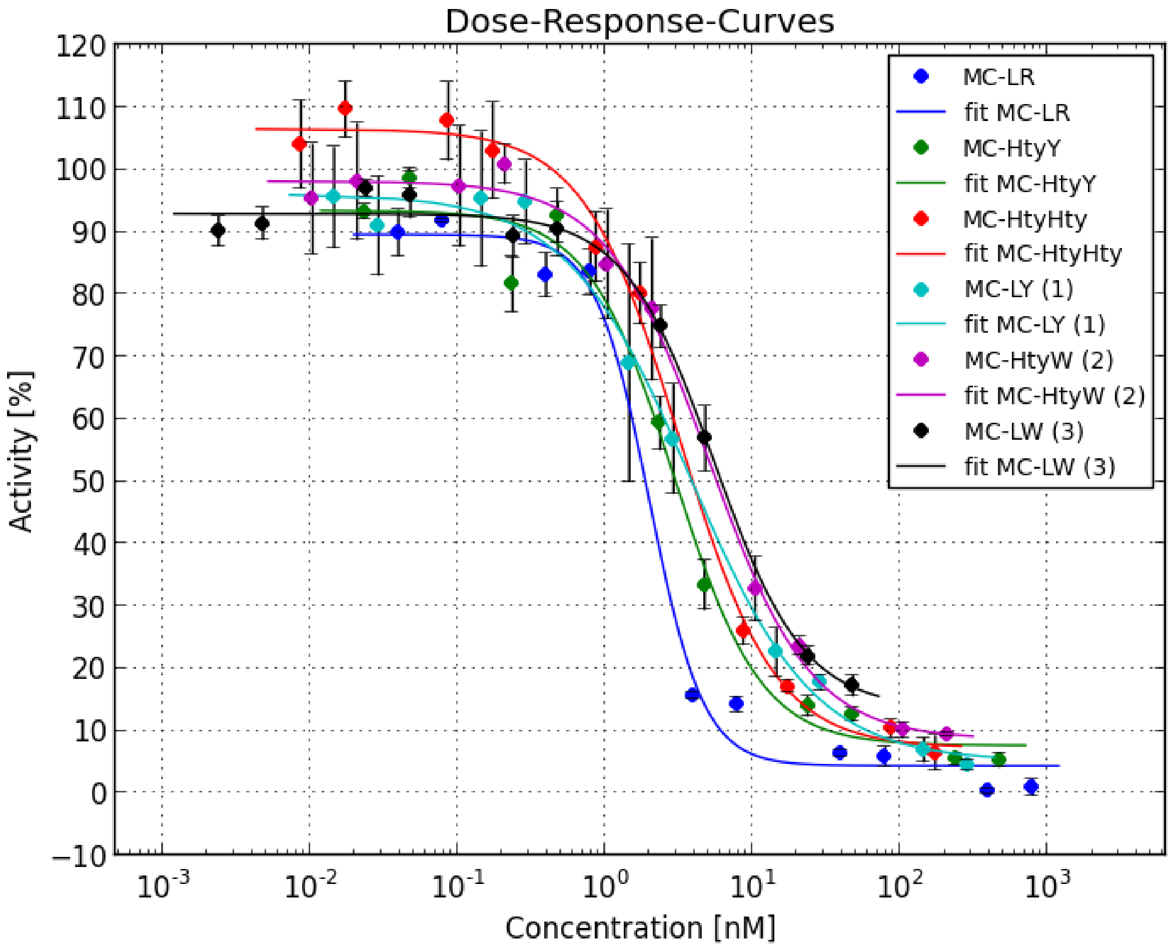

Dose-Response-Curves
Activity [%]
Concentration [nM]
120
110
100
90
80
70
60
50
40
30
20
10
0
−10
10^{-3}
10^{-2}
10^{-1}
10^{0}
10^{1}
10^{2}
10^{3}
MC-LR
fit MC-LR
MC-HtyY
fit MC-HtyY
MC-HtyHty
fit MC-HtyHty
MC-LY (1)
fit MC-LY (1)
MC-HtyW (2)
fit MC-HtyW (2)
MC-LW (3)
fit MC-LW (3)

Typical UV spectra of microcystins

Microcystin containing no aromatic amino acid (e.g. MC-LR)

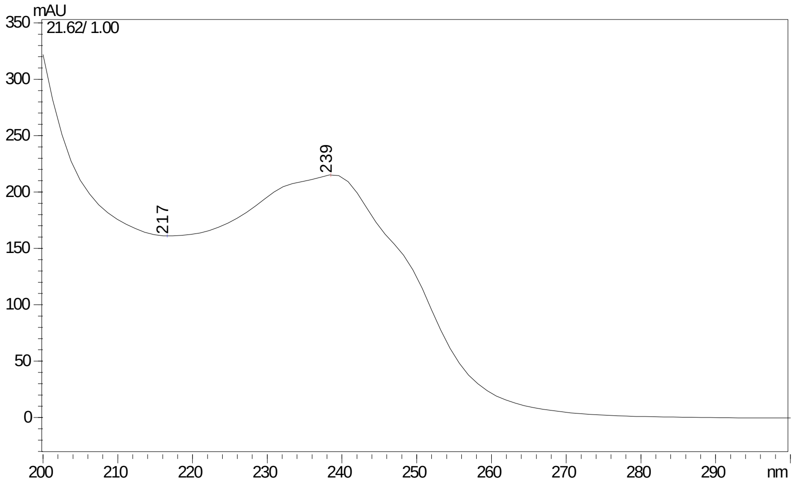

Microcystins containing aromatic amino acids such as Tyr or Hty (e.g. compound 1)

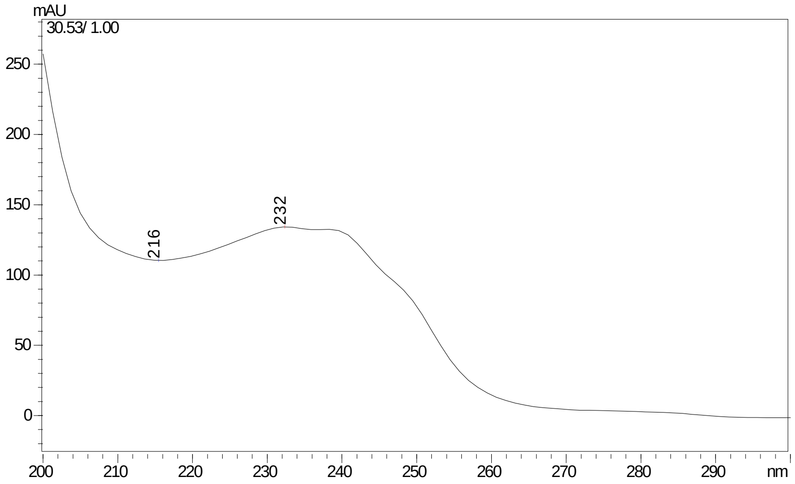

Microcystins containing Trp (e.g. compound 2)

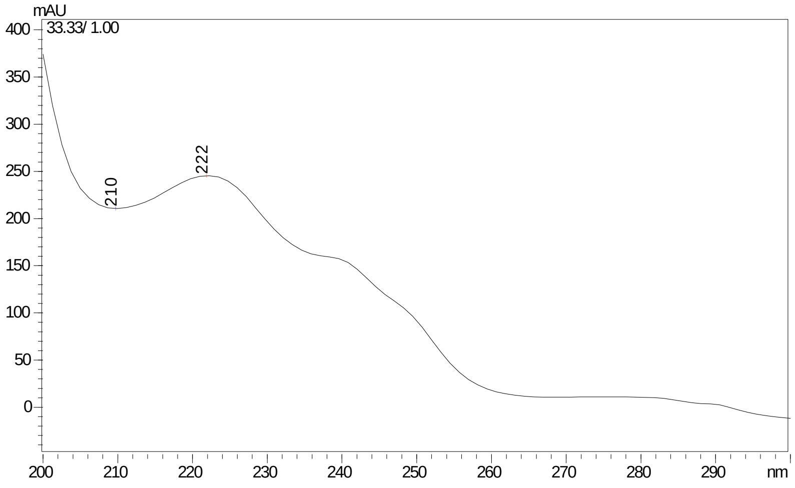

Supplement: Supplementary file 1 — All NMR and MS raw data as well as the Python script used to calculate the composition of possible MC congeners are available free of charge for download at http://dx.doi.org/10.6084/m9.figshare.706367. Detailed evaluation and annotation of the tandem mass spectra, 1H NMR spectra, output of the Python script, and information on the IC50 calculation, are available in the online version of this article. (PDF 8671 kb) [file 13659_2013_1_MOESM1_ESM.pdf]
